# Supplementary material for: Modeling the epidemiological impact of the UNAIDS 2025 targets to end AIDS as a public health threat by 2030
Source: PLoS Med. 2021 Oct 18;18(10):e1003831. doi: 10.1371/journal.pmed.1003831 (PMC8559943; doi:10.1371/journal.pmed.1003831)
Supplement: S1 Text — ASM, Age-Structured Model. (DOCX) [file pmed.1003831.s002.docx]

Goals ASM: An Age-structured Goals Model

Avenir Health, Glastonbury CT, USA

# Introduction

We developed an age-structured Goals model, Goals ASM, to project HIV epidemic indicators and evaluate programmatic impact of HIV programs prioritized by age in generalized epidemic settings. This annex describes the Goals ASM model structure, input assumptions, and scenarios used to evaluate the impact of achieving new UNAIDS targets for 2025.

# Model Structure

Goals ASM extends a previously published model that simulates heterosexual HIV transmission stratified by sex and age [[1](#_ENREF_1)]. The model resides in the Spectrum software suite and uses the same demographic structure and dynamics of HIV disease progression and mortality, antiretroviral treatment (ART) uptake and retention as the AIDS Impact Module (AIM) in Spectrum, which are detailed elsewhere [[2](#_ENREF_2), [3](#_ENREF_3)]. The novel components of Goals ASM are 1) its mechanistic representation of sexual mixing and HIV transmission by age, 2) representation of HIV interventions prioritized to the general population or key populations (female sex workers (FSW), men who have sex with men (MSM), and people who inject drugs (PWID)), and 3) its calibration to HIV prevalence data from household surveys and among antenatal clinics (ANC) clients. This annex describes these components in detail.

## HIV infection stages and treatment

AIM, and hence Goals ASM, stratifies adults (ages 15 and up) living with HIV into seven infection stages defined by CD4 cell count thresholds (>500, 350-500, 250-349, 200-249, 100-199, 50-99, <50 cells/mm^3^) and time on ART (not on ART, on ART for <6 months, 6-12 months, or >12 months). Goals ASM assumes the risk of HIV transmission varies by stage of infection [[4](#_ENREF_4), [5](#_ENREF_5)] and is lower for people on ART [[6](#_ENREF_6)] with suppressed viremia.

Goals ASM models reductions in HIV-related mortality on ART as more ART patients are viral suppressed. AIM takes as input HIV-related mortality rates for patients on ART $\mu_{s,a,h,d}^{\mathrm{ART}}\left( t \right)$ that are stratified by sex $s$, age $a$, infection stage $h$ at ART initiation, time on ART $d$, and time $t$ [[7](#_ENREF_7)]. Goals ASM simulates reductions in HIV-related mortality on ART by multiplying inputs $\mu_{s,a,h,d}^{\mathrm{ART}}\left( t \right)$ by a factor $\tilde{\mu}_{s,a}\left( t \right)$ that depends on the proportion of ART patients who are virally suppressed $v_{s,a}\left( t \right)$:

$\tilde{\mu}_{s,a}\left( t \right)=\min\left\{ 1, 1-0.5\cdot\frac{v_{s,a}\left( t \right)-v_{s,a}\left( \tau\right)}{0.95-v_{s,a}\left( \tau\right)} \right\}$ (1)

Here $\tau$ is the first year of model projection, typically $\tau=1970$, so that $v_{s,a}\left( \tau\right)$ is the earliest viral suppression inputted into the model. Since $v_{s,a}\left( t \right)$ is conditional on being on ART, the input has no effect on model outcomes before ART is available. Most countries have only routinely reported viral suppression data to UNAIDS for a few recent years. We typically assume viral suppression levels prior to data availability were constant at the earliest levels reported. Thus, mortality on ART in Goals ASM decreases linearly as viral suppression increases over initial levels, then stabilizes once 95% of ART patients are virally suppressed.

## HIV transmission

Individuals of sex $s$ in Goals ASM may debut sexually from age $a_{s,0}$ onward ($a_{s,0}=15$ in the present analysis). The model calculates HIV incidence $\lambda_{s,a}(t)$ among adults by age $a\in\{a_{s,0},\ldots,79,80+\}$ and sex $s$ over time $t$ according to the following formula

$\lambda_{s,a}\left( t \right)=\sum_{b=15}^{80} c_{s,a,b}^{*} (t)\cdot\varphi_{s,a}(t)\cdot\gamma_{s,a,b}(t)\sum_{h=1}^{7} \sum_{d=1}^{4} \beta_{s,b,h,d}\left( t \right)\cdot Y_{z,b,h,d}(t)/N_{z,b}(t)$ (2)

The constituent terms are:

- $Y_{z,b,h,d}$ is the number of people living with HIV of sex $z$ opposite to $s$ and age $b$ in infection stage $h$ and ART status $d$.
- $N_{z,b}$ is the total number of people of opposite sex $z$ and age $b$.
- $c_{s,a,b}^{*}$ denotes the rate that a person of sex $s$ and age $a$ acquires opposite-sex partners of age $b$.
- $\beta_{s,b,h,d}$ denotes the probability of HIV acquisition per partnership by an HIV negative partner of sex $s$ from an opposite-sex HIV positive partner of age $b$ in infection stage $h$ and ART status $d$.
- $\gamma_{s,a,b}$ denotes the effects of sexually transmitted infections and biomedical HIV prevention methods on HIV acquisition.
- $\varphi_{s,a}$ is the relative risk of HIV acquisition due to key population membership.

Population sizes $Y_{s,a,h,d}$ and $N_{s,a}$ are summed directly from compartments in AIM. The remaining terms are described in more detail in the listed order above.

**Partner change rates.** Partner change rates $c_{s,a,b}^{*}(t)$ are balanced to account for partnership supply and demand from each sex [[8](#_ENREF_8), [9](#_ENREF_9)]. This balancing accounts for “nominal” partner change rates $c_{s,a}(t)$, population sizes $N_{s,a}(t)$, mixing coefficients $m_{s,a,b}$, and balancing terms $\Delta_{s,a,b}\left( t \right).$ Mixing coefficients $m_{s,ab}$ specify the extent that sex $s$ age $a$ people mix with age $b$ opposite-sex partners. We define

$c_{s,a,b}^{*}\left( t \right)=c_{s,a}\left( t \right)\cdot m_{s,a,b}\cdot\Delta_{s,a,b}\left( t \right)$ (3)

Balancing terms $\Delta_{s,a,b}$ are calculated dynamically to satisfy the balance equation between females ($s=F$) and males ($s=M)$ for all ages $15\leq a,b\leq80$:

$c_{F,a}\left( t \right)\cdot m_{F,a,b}\cdot\Delta_{F,a,b}\left( t \right)\cdot N_{F,a}\left( t \right)=c_{M,b}\left( t \right)\cdot m_{M,b,a}\cdot\Delta_{M,b,a}\left( t \right)\cdot N_{M,b}\left( t \right)$ (4)

We solve this equation by setting $\Delta_{F,a,b}=1$, which implies

$\Delta_{M,b,a}(t)=\left[ c_{F,a}\left( t \right)\cdot m_{F,a,b}\cdot N_{F,a}\left( t \right) \right]/\left[ c_{M,b}\left( t \right)\cdot m_{M,b,a}\cdot N_{M,b}\left( t \right) \right]$ (5)

Substituting this into Eqn. 3 simplifies the balanced partner change rate for males,

$c_{M,a,b}^{*}=c_{F,b}\left( t \right)\cdot m_{F,b,a}\cdot N_{F,b}\left( t \right)/N_{M,a}\left( t \right)$ (6)

This shows that nominal partner change rates $c_{M,a}\left( t \right)$ and mixing coefficients $m_{M,a,b}$ for males do not need to be specified, since these are implicit in the corresponding parameters for females.

Nominal partner change rates $c_{F,a}\left( t \right)$ for females are specified in Goals ASM as the product of a trend in average numbers of lifetime partners $L\left( t \right)$ and age-specific rate ratios $c(a)$,

$c_{F,a}\left( t \right)=L\left( t \right)\cdot c(a)$ (7)

Lifetime partner trends can be entered into Goals ASM year-by-year or using a logistic curve $L\left( t;\theta\right)$

$L\left( t;\theta\right)=\theta_{1}+\frac{\theta_{2}-\theta_{1}}{\left[ 1+\exp\left( -\theta_{3}\left[ t-\tau-\theta_{4} \right] \right) \right]^{1/\theta_{5}}}$ (8)

The parameter vector $\theta=\left( \theta_{1},\theta_{2},\theta_{3},\theta_{4},\theta_{5} \right)$ controls the initial value $\theta_{1}$, final value $\theta_{2}$, growth rate $\theta_{3}$, years to final value $\theta_{4}$ and growth location $\theta_{5}$ of the lifetime partner curve. The time $\tau$ is the first year of the model projection, typically $\tau=1970$.

The age-specific rate ratios $c\left( a \right)$ are defined using a lognormal distribution density that has been shifted to start at age 15,

$c\left( a;\mu,\sigma^{2} \right)=\left\{ \begin{matrix} \frac{1}{(a-15)\sigma\sqrt{2\pi}}e^{-\left( \ln\left( a-15 \right)-\mu\right)^{2}/(2\sigma^{2})}, & a>15 \\ 0, & a\leq15 \end{matrix} \right.$ (9)

The location and scale parameters $\mu$ and $\sigma^{2}$ are not entered into Goals ASM directly. Instead, the model takes as input the age of peak sexual activity ($a_{\mathrm{peak}}$) and age by which half of lifetime partners are acquired on average ($a_{\mathrm{half}}$). We derive the location and scale parameters from these via $\mu=ln(a_{\mathrm{half}}-15)$ and $\sigma^{2}=\ln\left( \left[ a_{\mathrm{half}}-15 \right]/\left[ a_{\mathrm{peak}}-15 \right] \right)$.

Partner change rates in Goals ASM are typically adjusted during model fitting to calibrate the model to HIV prevalence data. Calibrated lifetime numbers of partners values may be higher than reported in nationally representative household surveys as those surveys may not include populations at high HIV risk, such as female sex workers, while these groups are implicit in the modelled population.

**Mixing coefficients.** Mixing coefficients for females $m_{F,a,b}$ are specified from the age difference $a-b$ between partners using a normal distribution,

$m_{F,a,b}\propto\exp\left( -\frac{\left( b-a-\mu_{m} \right)^{2}}{2\sigma_{m}^{2}} \right)$ such that $\sum_{b=15}^{80} m_{F,a,b}=1$ (10)

The mean ($\mu_{m}$) and variance ($\sigma_{m}^{2}$) in partner age differences can be entered into Goals ASM directly or calibrated during model fitting. We assume these parameters do not vary as females age, based on analysis of partner age differences reported by Demographic and Health Survey (DHS) respondents in sub-Saharan Africa (unpublished Avenir Health analysis).

**HIV transmission probabilities**. Goals ASM calculates HIV transmission probabilities $\beta_{s,b,h,d}$ on a per-partnership basis. These probabilities depend on the sex $s$ of the HIV negative partner, and the age $b$, infection stage $h$, and ART status $d$ of the HIV positive partner. We denote the sex of the HIV positive partner as $z$ below.

$\beta_{s,b,h,d}=\beta\cdot\beta_{s}\cdot\left\{ \begin{matrix} \beta_{z,b,h}(t), & d: on ART<6 mos or off ART \\ \nu_{z,b}(t)\cdot\left( 1-\varepsilon^{\mathrm{VS}} \right)+\left( 1-\nu_{z,b}(t) \right)\cdot\beta_{z,b,h}\left( t \right), & d: on ART\geq6 mos \end{matrix} \right.$ (11)

The transmission probability depends on a “baseline” probability $\beta$ modulated by a sex-specific factor $\beta_{s}$. We assume $\beta_{M}=1$ but allow $\beta_{F}\geq1$ to account for any excess risk of male-to-female HIV transmission not captured by other modeled mechanisms [[10](#_ENREF_10), [11](#_ENREF_11)]. HIV positive partners who are not on ART, who have been on ART less than 6 months, or who have been on ART at least six months but have unsuppressed viremia (proportion $1-\nu_{z,b}(t)$) have transmission risk that depends on their infection stage according to a time-varying multiplier $\beta_{z,b,h}\left( t \right)$ described below. Viral suppression on ART reduces HIV transmission by a factor of $\varepsilon^{\mathrm{VS}}$; in keeping with evidence that HIV is untransmissible with undetectable viral loads [[6](#_ENREF_6), [12-14](#_ENREF_12)], we used $\varepsilon^{\mathrm{VS}}=1$ in the present analyses.

AIM does not include a compartment for primary HIV infection stage. People who newly acquire HIV may start out in any of the model’s seven CD4-based infection stages. Therefore, Goals ASM supposes some PLHIV may have primary infection in any infection stage. We define transmission multipliers for ($\tilde{\beta}_{P}$) or symptomatic ($\tilde{\beta}_{S})$ infection relative to asymptomatic infection ($\tilde{\beta}_{A}=1$), then calculate $\beta_{h}\left( t \right)$ as follows:

$\beta_{z,b,h}\left( t \right)=\left\{ \begin{matrix} \tilde{\beta}_{P}\cdot p_{z,b,h}\left( t \right)+\tilde{\beta}_{A}\cdot\left( 1-p_{z,b,h}\left( t \right) \right), & CD4\geq200 \mathrm{cells}/\mathrm{mm}^{3} (h\in\left\{ 1,2,3,4 \right\}) \\ \tilde{\beta}_{P}\cdot p_{z,b,h}\left( t \right)+\tilde{\beta}_{S}\cdot\left( 1-p_{z,b,h}\left( t \right) \right), & CD4<200 \mathrm{cells}/\mathrm{mm}^{3} (h\in\left\{ 5,6,7 \right\}) \end{matrix} \right.$ (12)

We calculate the proportion of PLHIV with primary infection, $p_{z,b,h}\left( t \right)$, based on the proportion of people who enter stage $h$ at infection ($\pi_{z,b,h}$), the duration of primary infection in months $w$, and the proportion of people in stage $h$ who were newly infected. We approximate this latter proportion as the ratio between incident infections $\pi_{z,b,h}\cdot I_{z,b}(t-1)$ and prevalent infections $\sum_{d=1}^{4} Y_{z,b,h,d}(t-1)$ in that stage in the previous year:

$p_{z,b,h}\left( t \right)=\left( w/{12} \right)\cdot\pi_{z,b,h}\cdot I_{z,b}(t-1)/\sum_{d=1}^{4} Y_{z,b,h,d}(t-1)$ (13)

The baseline transmission probability $\beta$ and sex-specific factor $\beta_{s}$ were calibrated during model fitting for each country. We assumed transmission multipliers $\tilde{\beta}_{P}=26$ and $\tilde{\beta}_{S}=7$ and a primary infection duration of $w=2.9$ months in all countries [[4](#_ENREF_4)].

**Effects of STIs and biomedical HIV prevention method.** The term $\gamma_{s,a,b}$ in Equation 2 encapsulates factors that may increase (sexually transmitted infections) or decrease (biomedical HIV prevention methods) the risk of sexual HIV acquisition by HIV negative individuals of sex $s$ and age $a$ from age $b$ opposite-sex partners:

$\gamma_{s,a}=\left( 1+\kappa_{s,a}^{\mathrm{STI}}(t)\cdot\varepsilon_{s}^{\mathrm{STI}} \right)\left( 1-\sum_{i} \kappa_{s,a,i}^{\mathrm{PrEP}}\left( t \right)\cdot\varepsilon_{s,a,i}^{\mathrm{PrEP}} \right)\left( 1-\kappa_{s,a}^{\mathrm{MC}}\left( t \right)\cdot\varepsilon^{\mathrm{MC}} \right)\left( 1-\kappa_{s,a,b}^{\mathrm{Cndm}}\left( t \right)\cdot\varepsilon^{\mathrm{Cndm}} \right)$ (14)

Each factor in Equation 14 consists of an intervention coverage or STI prevalence term $\kappa$ and effect $\varepsilon$.

- **STIs:** STIs magnify the modeled risk of HIV acquisition based on age- and sex-specific prevalence $\kappa_{s,a}^{\mathrm{STI}}\left( t \right)$ and transmission multiplier $\varepsilon_{s}^{\mathrm{STI}}$. STI prevalence was based on reported STI symptoms (reported STI, genital discharge, sores or ulcers) in their most recent DHS [[15](#_ENREF_15)] or on national data or regional estimates of HSV-2 prevalence [[16](#_ENREF_16)]. We assumed STIs increase the risk of HIV acquisition three-fold ($\varepsilon_{s}^{\mathrm{STI}}=3$) [[17-19](#_ENREF_17)].
- **Pre-exposure prophylaxis (PrEP):** Goals ASM represents a mix of oral, injectable, vaginal gel, and vaginal ring PrEP methods ($i\in\{1,2,3,4\}$). We specify time-varying coverage levels $\kappa_{s,a,i}^{\mathrm{PrEP}}\left( t \right)$ and static effectiveness levels $\varepsilon_{s,a,i}^{\mathrm{PrEP}}$ for each sex $s$, age $a$, and method $i$. Coverage levels of vaginal PrEP methods cannot be specified for males. Method effectiveness is the product of two sex- and age-stratified inputs ($\varepsilon_{s,a,i}^{\mathrm{PrEP}}=\alpha_{s,a,i}^{\mathrm{PrEP}}\cdot\eta_{s,a,i}^{\mathrm{PrEP}})$: adherence to regular method use ($0\leq\alpha\leq1$), and biological efficacy of each method ($0\leq\eta\leq1$). In the present exercise we assumed 71% adherence to 99% efficacious oral PrEP [[20-22](#_ENREF_20)] and no coverage of non-oral PrEP methods.
- **Male circumcision:** We assume male circumcision reduces HIV acquisition by 60% ($\varepsilon^{\mathrm{MC}}=0.6$) [[23-25](#_ENREF_23)]. We based male circumcision prevalence $\kappa_{s,a}^{\mathrm{MC}}\left( t \right)$ on estimates from Demographic and Health Surveys, AIDS Indicator Surveys, and/or Population-based HIV Impact Assessment surveys in most countries. In countries that have been scaling up voluntary medical male circumcision (VMMC), we fitted age-specific male circumcision prevalence trends to age-specific estimates from each available survey. In countries without VMMC scale-up we assumed male circumcision prevalence remained constant over time at levels measured in the latest national household survey. For notational convenience Equation 14 includes a prevalence term $\kappa_{F,a}^{\mathrm{MC}}\left( t \right)$ for females, though we do not model female genital mutilation ($\kappa_{F,a}^{\mathrm{MC}}\left( t \right)=0$).
- **Condom use:** Condom use is specified by the frequency of condom use at last sex $\kappa_{s,a,b}^{\mathrm{Cndm}}\left( t \right)$, which is assumed to reduce HIV acquisition 80% ($\varepsilon^{\mathrm{Cndm}}=0.8)$ [[26](#_ENREF_26)]. We input age-specific, sex-agnostic condom use frequencies $\tilde{\kappa}_{a}\left( t \right)$ into Goals ASM, then base condom use frequency within heterosexual partnerships on the male partner’s age ($\kappa_{F,a,b}^{\mathrm{Cndm}}\left( t \right)=\tilde{\kappa}_{b}(t)$ and $\kappa_{M,a,b}^{\mathrm{Cndm}}\left( t \right)=\tilde{\kappa}_{a}(t)$). In countries with nationally representative household surveys we fitted age-stratified condom use trends to data on condom use at last sex from male and female respondents.

**Key population interventions.** Key populations (FSW, MSM, and PWID) are not explicitly stratified into compartments in Goals ASM for two reasons. First, the model uses the existing compartmental structure of AIM, which does not include behavioral risk stratifications. Second, critical data to inform key population modeling (population size and HIV prevalence estimates) are sparse, not nationally representative, or both in most of sub-Saharan Africa [[27-29](#_ENREF_27)]. The model approximates the impact of HIV prevention interventions prioritized to key populations by applying an adjustment factor $\varphi_{s,a}\left( t \right)$ to overall incidence by age and sex. This is the product of “impact” factors related to PrEP use ($\varphi_{r,a}^{\mathrm{PrEP}}$) and changes in condom use ($\varphi_{r,a}^{\mathrm{Cndm}}$) or safe injection practices ($\varphi_{r,a}^{\mathrm{SI}}$) by risk group $r$:

$\varphi_{s,a}\left( t \right)=\left\{ \begin{matrix} \varphi_{\mathrm{FSW},a}^{\mathrm{PrEP}}\left( t \right)\cdot\varphi_{\mathrm{FWID},a}^{\mathrm{PrEP}}\left( t \right)\cdot\varphi_{FSW,a}^{\mathrm{Cndm}}\left( t \right)\cdot\varphi_{\mathrm{FWID},a}^{\mathrm{Cndm}}\left( t \right)\cdot\varphi_{\mathrm{FWID},a}^{\mathrm{SI}}\left( t \right), & s=F \\ \varphi_{\mathrm{MSM},a}^{\mathrm{PrEP}}\left( t \right)\cdot\varphi_{\mathrm{MWID},a}^{\mathrm{PrEP}}\left( t \right)\cdot\varphi_{MSM,a}^{\mathrm{Cndm}}\left( t \right)\cdot\varphi_{\mathrm{MWID},a}^{\mathrm{Cndm}}\left( t \right)\cdot\varphi_{\mathrm{MWID},a}^{\mathrm{SI}}\left( t \right), & s=M \end{matrix} \right.$ (15)

Using $Z\in\{\mathrm{PrEP}, \mathrm{Cndm}, SI\}$ as shorthand, each impact factor $\varphi_{r,a}^{Z}$ accounts for the contribution of key population $r$ to incidence in the broader same-sex population aged $a$. We calculate these based on inputs $n_{r,a}\left( t \right)$ and $\rho_{r,a}(t)$ that specify the population proportion and relative incidence rate in key population $r$ relative to the broader same-sex population aged $a$:

$\varphi_{r,a}^{Z}\left( t \right)=\frac{1-n_{r,a}\left( t \right)+n_{r,a}\left( t \right)\cdot\rho_{r,a}\left( t \right)\cdot\psi_{r,a}^{Z}(t)}{1-n_{r,a}\left( t \right)+n_{r,a}\left( t \right)\cdot\rho_{r,a}\left( t \right)}$ (16)

The terms $\psi_{r,a}^{Z}(t)$ are structured differently for each program area. One commonality across areas is that impact of key population programs is calculated based on changes in coverage since a specified base-year $t_{0}$ ($t_{0}=2019$ in the present analyses). The effects of key population programs in earlier years are assumed implicit in modeled incidence before then, so that earlier coverage levels do not need to be collected.

- **PrEP**: As with the more general population above, key population members may access a mix of PrEP methods (oral, injectable, gel, or ring). The factor $\psi_{r,a}^{Z}(t)$ accounts for coverage $\kappa_{r,a}^{\mathrm{PrEP}}\left( t \right)$, adherence ($\alpha_{r,a,i}^{\mathrm{PrEP}})$ and efficacy ($\eta_{r,a,i}^{\mathrm{PrEP}}$) of each PrEP method $i$:

$\psi_{r,a}^{\mathrm{PrEP}}\left( t \right)=\left\{ \begin{matrix} \max\left\{ 0, 1-\sum_{i} \left[ \kappa_{r,a,i}^{\mathrm{PrEP}}\left( t \right)-\kappa_{r,a,i}^{\mathrm{PrEP}}\left( t_{0} \right) \right]\cdot\alpha_{r,a,i}^{\mathrm{PrEP}}\cdot\eta_{r,a,i}^{\mathrm{PrEP}}) \right\}, & t\geq t_{0} \\ 1, & t<t_{0} \end{matrix} \right.$ (17)

We assume no PrEP coverage at baseline $t=t_{0}$ in the present exercise.

- **Condom use**: Goals ASM requires that condom use at last sex $\kappa_{r,a}^{\mathrm{Cndm}}(t)$ be specified for each key population. Key population outreach programs with coverage $\kappa_{r,a}^{\mathrm{OR}}(t)$ and effect $\varepsilon_{r,a}^{\mathrm{OR}}$ may promote condom use. This is modeled as a reduction in condom non-use [[30](#_ENREF_30)],

$\kappa_{r,a}^{\mathrm{Cndm}}\left( t \right)=1-\left( 1-\kappa_{r,a}^{\mathrm{Cndm}}\left( t_{0} \right) \right)\left( 1+\kappa_{r,a}^{\mathrm{OR}}\left( t \right)\cdot\varepsilon_{r,a}^{\mathrm{OR}} \right)$ (18)

This calculation feeds into the term $\psi_{r,a}^{\mathrm{Cndm}}\left( t \right)$ for condom use,

$\psi_{r,a}^{\mathrm{Cndm}}\left( t \right)=\left\{ \begin{matrix} \max\left\{ 0, 1-\left[ \kappa_{r,a}^{\mathrm{Cndm}}\left( t \right)-\kappa_{r,a}^{\mathrm{Cndm}}\left( t_{0} \right) \right]\cdot\varepsilon^{\mathrm{Cndm}} \right\}, & t\geq t_{0} \\ 1, & t<t_{0} \end{matrix} \right.$ (19)

- **Safe infection practices:** Goals ASM includes three behavior change programs that may reduce needle sharing or other unsafe injecting behavior among PWID [[31-33](#_ENREF_31)]: outreach programs ($\mathrm{OR}$), needle-syringe exchange programs (NSEP), and opioid substitution therapy (OST). The model takes the coverage $\kappa_{r,a,i}^{\mathrm{SI}}\left( t \right)$ of each program, its effect on needle sharing $\varepsilon_{r,a,i}^{\mathrm{SI},1}$, and its effect on other unsafe injection practices $\varepsilon_{r,a,i}^{\mathrm{SI},2}$ as input and calculates program effects multiplicatively:

$\zeta_{r,a}^{\mathrm{SI}}\left( t \right)=\prod_{i\in\{OR, NSEP,OST\}} \prod_{j=1}^{2} \left( 1+\left[ \kappa_{r,a,i}^{\mathrm{SI}}\left( t \right)-\kappa_{r,a,i}^{\mathrm{SI}}\left( t_{0} \right) \right]\cdot\varepsilon_{r,a,i}^{\mathrm{SI},j} \right)$ (20)

Ultimately, the impact factor for safe injection practices $\psi_{r,a}^{\mathrm{SI}}\left( t \right)$ is implemented as a reduction to the proportion $u_{s}\left( t \right)$ of PWID who share needles or syringes:

$\psi_{r,a}^{\mathrm{SI}}\left( t \right)=\left\{ \begin{matrix} \max\left\{ 0, 1-u_{s}\left( t_{0} \right)\cdot\left( 1-\zeta_{r,a}^{SI}\left( t \right) \right) \right\}, & t\geq t_{0} \\ 1, & t<t_{0} \end{matrix} \right.$ (21)

Goals ASM was applied in countries in sub-Saharan Africa only. We used country-specific key population inputs when they were available, otherwise we used regional estimates for either Western and Central Africa or Eastern and Southern Africa in constituent countries. We obtained population size estimates, base-year condom use, and base-year outreach program coverage estimates from the UNAIDS Key Population Atlas [[34](#_ENREF_34)]. We assumed 10% of PWID are female and that 25% of PWID share needles in the sub-Saharan African contexts modeled [[35](#_ENREF_35)]. We used published odds ratios for HIV prevalence among female sex workers relative to HIV prevalence in the female population [[36](#_ENREF_36)] as a proxy for relative incidence rate ratios. For lack of representative data, we assumed incidence rates in MSM and PWID were the same as in the general population of the same age and sex.

## Behavior change programs

Goals ASM models behavior change programs (BCP) outside key populations alongside biomedical interventions and key population programs. These may be prioritized by age, particularly to adolescent girls and young women (AGYW) or adolescent boys and young men (ABYM). These interventions include

- Condom promotion and provision
- Violence prevention and post-violence care
- HIV testing and counselling
- Social asset building
- Family planning
- Parenting and care giver programs
- Educational subsidies
- Cash transfers
- Economic empowerment
- School-based prevention and sexuality education
- Out of school programs
- Community mobilization and norms change

Goals ASM takes as input coverage trends $\kappa_{s,a,j}^{\mathrm{BCP}}(t)$ by sex and broad age group (15-24, 25-34, 35-44, 45+) for each program $j$ above, and a matrix of impact factors $\varepsilon_{s,a,j,k}^{\mathrm{BCP}}$ that quantify the effect behavior change program $j$ has on each of five outcomes $k$:

1. Numbers of sexual partners
2. The extent of age-disparate mixing
3. Condom use
4. Experience of partner or sexual violence
5. Age of sexual debut

School-based programs (school-based prevention and sexuality education, educational subsidies) are applied to ages 15-24 only. Coverage levels of these programs are calculated as the product of 1) input program coverage among school attendees, 2) the proportion of ages 15-24 who are secondary school ages (15-18), and 3) secondary school gross enrollment rates by sex. Meanwhile, coverage of out of school programs are calculated analogously by multiplying the input coverage level among out of school ages 15-18 by the proportion of ages 15-18 who are not enrolled in school.

We calculate the joint effect $\omega_{s,a,k}\left( t \right)$ of each program area on each outcome $k=\{1,2,3,4,5\}$. For the first four outcomes (numbers of sexual partners, condom use, experience of partner or sexual violence, age-disparate mixing) we use the formula

$\omega_{s,a,k}\left( t \right)=\left\{ \begin{matrix} \prod_{j} \left( 1+\left[ \kappa_{s,a,j}^{\mathrm{BCP}}\left( t \right)-\kappa_{s,a,j}^{\mathrm{BCP}}\left( t_{0} \right) \right]\cdot\varepsilon_{s,a,j,k}^{\mathrm{BCP}} \right), & t\geq t_{0} \\ 1, & t<t_{0} \end{matrix} \right. \mathrm{for} k\in\{1,2,3,4\}$ (22)

Whereas we anticipate behavior change interventions will decrease the frequency of risky behaviors for these first four outcomes, we model risk reductions due to older age at sexual debut ($k=5$), which necessitates a slightly different formula:

$\omega_{s,a,k}\left( t \right)=\left\{ \begin{matrix} \prod_{j} \left[ \kappa_{s,a,j}^{\mathrm{BCP}}\left( t \right)-\kappa_{s,a,j}^{\mathrm{BCP}}\left( t_{0} \right) \right]\cdot\varepsilon_{s,a,j,k}^{\mathrm{BCP}}, & t\geq t_{0} \\ 1, & t<t_{0} \end{matrix} \right. \mathrm{for} k=5$ (23)

As with key population programs above, we assume that the effects of behavior change programs before the intervention base-year $t_{0}$ ($t_{0}=2019$ in the present analysis) are implicit in the modelled incidence trend before $t_{0}$.

Below, we describe how behavior change programs affect terms in the Goals ASM transmission calculation. For example, suppose $f\left( t \right)$ is a term in the transmission calculation in the absence of behavior change programs, and suppose $\omega\left( t \right)$ is the effect behavior change programs have on that term. Then we write $f\left( t \right)\leftarrow f\left( t \right)\cdot\omega\left( t \right)$ to convey that in the presence of behavior change programs $f\left( t \right)\cdot\omega\left( t \right)$ replaces $f\left( t \right)$ in the transmission calculation.

**Numbers of sexual partners (**$\boldsymbol{k=1}$**)**. The joint effect $\omega_{s,a,1}\left( t \right)$ of behavior change interventions on partner change rates is used to update the balanced partner change rate $c_{s,a,b}^{*}\left( t \right)$ defined in Equation 3:

$c_{s,a,b}^{*}\left( t \right)\leftarrow c_{s,a,b}^{*}\left( t \right)\cdot\omega_{s,a,1}\left( t \right)\cdot\omega_{z,b,1}\left( t \right)$ (24)

We multiply $c_{s,a,b}^{*}\left( t \right)$ by both $\omega_{s,a,1}\left( t \right)$ and $\omega_{z,b,1}\left( t \right)$ to ensure that partnership supply and demand remain balanced between sexes, and to account for effects of behavior change programs that reach sex $s$ and age $a$ and programs that reach their potential opposite sex $z$ age $b$ partners. This imply programs that directly reduce partner change rates among women will indirectly reduce partner change rates among their potential male partners and vice versa.

**Age-disparate mixing (**$\boldsymbol{k=2}$**).** Behavior change programs may affect the proportion of 15-24 females who have sex with males five or more years older in Goals ASM. This reduction affects the balanced partner change rates $c_{F,a,b}^{*}\left( t \right)$ for females at ages $15\leq a\leq24$ and males at ages $b\geq a+5$, and is applied after direct effects of behavior change programs on numbers of sexual partners in Equation 24:

$c_{F,a,b}^{*}(t)\leftarrow c_{F,a,b}^{*}(t)\cdot\omega_{F,a,2}\left( t \right)$ (25)

**Condom use (**$\boldsymbol{k=3}$**).** Goals ASM models behavior change program effects on condom use $\omega_{s,a,3}\left( t \right)$ as a fold change in condom non-use:

$\kappa_{s,a,b}^{\mathrm{Cndm}}\left( t \right)\leftarrow1-\left( 1-\kappa_{s,a,b}^{\mathrm{Cndm}}\left( t \right) \right)\cdot\sqrt{\omega_{s,a,3}\left( t \right)\cdot\omega_{z,b,3}\left( t \right)}$ (26)

We multiply the frequency of condom non-use $\left( 1-\kappa_{s,a,b}^{\mathrm{Cndm}}\left( t \right) \right)$ by the geometric mean of changes in sex $s$ age $a$ ($\omega_{s,a,3}\left( t \right)$) and their opposite sex $z$ age $b$ partners ($\omega_{z,b,3}\left( t \right)$) since a change in condom use in one sex must be reflected condom use among their partners. We use the geometric mean to combine the effects of programs reaching each partner since negotiated condom use within sexual partnerships may modulate program effects.

**Experience of partner or sexual violence (**$\boldsymbol{k=4}$**).** Goals ASM assumes women who experience partner or sexual violence may reduce condom use. The effects of interventions that reduce violence rely on two additional model inputs: the proportions of females ($s=F$) who experience partner or sexual violence $V_{s,a}(t)$ and their risk of condom non-use $q_{s,a}(t)$ relative to women who do not experience violence. These effects ($\omega_{s,a,4}\left( t \right)$) are applied after the direct effects of programs on condom use per Equation 26:

$\kappa_{s,a,b}^{\mathrm{Cndm}}\left( t \right)\leftarrow\kappa_{s,a,b}^{\mathrm{Cndm}}\left( t \right)+\max\left\{ 0, \left( 1-\kappa_{s,a,b}^{\mathrm{Cndm}}\left( t \right) \right)\cdot V_{s,a}\left( t_{0} \right)\cdot\left( 1-\omega_{s,a,4}\left( t \right) \right)\cdot q_{s,a}\left( t \right) \right\}$ (27)

In the equation above, $V_{s,a}\left( t_{0} \right)\cdot\left( 1-\omega_{s,a,4}\left( t \right) \right)$ quantifies the reduction in experience of violence due to behavior change programs; this is assumed to reduce condom non-use ($1-\kappa_{s,a,b}^{\mathrm{Cndm}}\left( t \right)$) proportional to the relative risk of condom non-use $q_{s,a}\left( t \right)$.

**Age of sexual debut:** Behavior change programs in Goals ASM may increase the age at sexual debut,

$a_{s,0}\left( t \right)\leftarrow a_{s,0}\left( t \right)+\omega_{s,a,5}\left( t \right)$ (28)

People are assumed sexually inactive ($c_{s,a,b}^{*}\left( t \right)=0$) at ages $a<\lfloor a_{s,0}\left( t \right)\rfloor$. If $a_{s,0}\left( t \right)$ is not an integer, we assume that partner change rates at age $\lfloor a_{s,0}\left( t \right)\rfloor$ are reduced accordingly: for example, if behavior change programs increase the age of sexual debut from 15 to 16.4, we reduce partner change rates to zero at age 15 and decrease them by 40% at age 16.

# Model Calibration

## Overview

Goals ASM is calibrated using a Bayesian framework that incorporates prior distributions on several model inputs and a likelihood that synthesizes HIV prevalence data from household surveys and HIV testing among pregnant women attending antenatal clinics (ANC). We calibrate Goals ASM by maximizing the Bayesian posterior distribution, the product of the prior and likelihood, using the Nelder-Mead simplex method [[37](#_ENREF_37)]. This method yields point estimates of model parameters adjusted during calibration.

## Likelihood

We let $\theta$ denote the vector of model parameters that are varied during model calibration. The likelihood $L\left( \theta|D \right)$ of $\theta$ given HIV prevalence data $D=(D_{\mathrm{ANC}}, D_{\mathrm{AGE}})$ is the product of an age-based likelihood $L_{\mathrm{AGE}}\left( \theta| D_{\mathrm{AGE}} \right)$ and an ANC-based likelihood $L_{\mathrm{ANC}}(\theta|D_{\mathrm{ANC}})$:

$L\left( \theta| D \right)=L_{\mathrm{AGE}}\left( \theta| D_{\mathrm{AGE}} \right)\cdot L_{\mathrm{ANC}}(\theta|D_{\mathrm{ANC}})$ (29)

The age-based likelihood compares model HIV prevalence estimates to data from national HIV prevalence surveys, stratified by sex $s$and five-year age group $a$. These data consist of the number tested for HIV $n_{s,a}\left( t \right)$ by age and sex and the number who tested HIV positive $y_{s,a}\left( t \right)$ in a survey conducted at time $t$. These numbers are weighted to account for the clustered survey design. The likelihood is formulated by assuming the number who test positive is binomially distributed given the number tested and the model estimate of HIV prevalence $p_{s,a}\left( t;\theta\right)$:

$L_{\mathrm{AGE}}\left( \theta| D_{\mathrm{AGE}} \right)=\prod_{s,a,t} \left( \begin{matrix} n_{s,a}(t) \\ y_{s,a}(t) \end{matrix} \right)\left[ p_{s,a}\left( t;\theta\right) \right]^{y_{s,a}(t)}\left[ 1-p_{s,a}\left( t;\theta\right) \right]^{n_{s,a}(t)-y_{s,a}(t)}$ (30)

The product above ranges over sex $s$, five-year age groups $a$, and survey time points $t$.

The ANC-based likelihood uses data on HIV prevalence in pregnant women attending antenatal clinics. These data are routinely used by countries in sub-Saharan Africa to estimate HIV incidence using the Estimation and Projection Package (EPP) [[38](#_ENREF_38)], and aggregate count data are published in Spectrum files available from UNAIDS [[39](#_ENREF_39)]. These include three types of data: site-level sentinel surveillance data, site-level routine testing data, and census-level routine testing data. Sentinel surveillance data consist of unlinked anonymous testing of pregnant women at selected surveillance sites. Since 2015, most countries have transitioned to use of routine HIV testing data among pregnant women per recommendations from UNAIDS and WHO [[40](#_ENREF_40), [41](#_ENREF_41)]. Countries use routine testing data collected at former sentinel surveillance sites to continue data time series for those sites, as well as census-level data aggregated across all women whose HIV status was ascertained during ANC care.

Sentinel surveillance data consist of numbers $n_{i,t}^{\mathrm{SS}}$ of pregnant women who were tested for HIV by facility $i$ and year $t$, and the number $y_{i,t}^{\mathrm{SS}}$ of pregnant women among them who tested HIV positive. Site-level routine testing data are analogous, but numbers $n_{i,t}^{\mathrm{RT}}$ and $y_{i,t}^{\mathrm{RT}}$ include pregnant women newly tested plus women who were known HIV positive at the first ANC visit of their current pregnancy. Census-level routine testing data consist of numbers ascertained $n_{t}^{\mathrm{RT}}$ and numbers ascertained as HIV positive $y_{t}^{\mathrm{RT}}$ nationally.

The likelihood model used to fit Goals ASM was developed for EPP, and full details of the likelihood specification have been reported previously [[42-44](#_ENREF_42)]. Like the household survey data above, it is derived from a binomial model, but includes clinic random effects for each site. The likelihood model includes clinic random effects for each site to account for purposive sampling of facilities, calibration terms to adjust for the transition from sentinel surveillance to routine testing in persistent ANC sites, and differences between pregnant women attending ANC and pregnant women overall, and variance inflation terms to account for non-sampling errors in ANC data. There are two key differences between the use of ANC data in Goals ASM in contrast to EPP. First, countries that use EPP for their incidence estimates typically subdivide the country into urban and rural epidemics, or into administrative regions, and subdivide ANC data based on facility location; by contrast, Goals ASM does not include these geographic strata, so we use combined ANC data across all EPP regions. Second, EPP previously calculated the ANC data likelihood based on differences between observed HIV prevalence at ANC and modeled prevalence in the 15-49 general population, whereas Goals ASM calculates the likelihood based on differences between observed prevalence at ANC and modeled prevalence in pregnant women, adjusted for HIV-related fertility effects by age, CD4 cell count, and ART status. This is consistent with use of these data in the more recent, age-structured version of EPP [[38](#_ENREF_38)].

## Prior distribution

The model can be calibrated by adjusting any combination of several input parameters. The joint prior distribution on these inputs consists of independent marginal prior distributions on each selected parameter. We specify uniform distributions for most of these inputs (Table 1). Not all inputs were fitted in each country in the present exercise. For example, inputs pertaining to the likelihood of observing ANC data were not fitted in countries without those data.

The modeling software allows several other inputs to be adjusted (epidemic start year, effects of STIs on HIV acquisition, STI prevalence trends, age of sexual debut, fertility rate ratio for HIV-positive women) in addition to those listed in Table 1, but these were not used to calibrate models in the present analysis.

Table 1. Prior distributions on model parameters

| Parameter | Symbol | Prior | # countries^1^ |
| --- | --- | --- | --- |
| HIV transmission probability per partnership, female to male | $\beta$ | Uniform(10%, 70%) | 39 |
| Odds ratio of male to female HIV transmission^2^ | $\xi$ | Uniform(1, 5) | 39 |
| Lifetime number of sexual partners |  |  |  |
| Initial value | $\theta_{1}$ | Uniform(1, 50) | 39 |
| Final value | $\theta_{2}$ | Uniform(1, 50) | 39 |
| Growth rate | $\theta_{3}$ | Uniform(0.0, 0.5) | 39 |
| Years to final value | $\theta_{4}$ | Uniform(10, 40) | 39 |
| Age of peak sexual activity | $a_{\mathrm{peak}}$ | Uniform(15, 35) | 38 |
| Age of median sexual activity^3^ | $\tilde{a}_{\mathrm{half}}$ | Uniform(0, 0.75) | 38 |
| Age difference between male and female sexual partners^4^ |  |  |  |
| Mean age difference | $\mu_{m}$ | Uniform(0, 10) | 15 |
| Variance | $\sigma_{m}^{2}$ | Uniform(1.5, 32) | 15 |
| ANC likelihood parameters^5^ |  |  |  |
| Site-level sentinel surveillance calibration term |  | Normal(0.15, 1.0) | 38 |
| Site-level routine testing calibration term |  | Normal(0.00, 1.0) | 35 |
| Census-level routine testing calibration term |  | Normal(0.00, 0.25^2^) | 29 |
| Site-level variance inflation term |  | Exponential(0.015) | 38 |
| Census-level variance inflation term |  | Exponential(0.015) | 29 |

^1^ Models were calibrated for 39 countries.

^2^ We use the odds ratio $\xi$ to adjust the relative risk of male-to-female transmission $\beta_{F}$, $\beta_{F}=\beta\cdot\xi/(1-\beta+\beta\cdot\xi)$.

^3^ We specify $\tilde{a}_{\mathrm{half}}$ as a relative quantity, then calculate absolute age $a_{\mathrm{half}}=a_{\mathrm{peak}}+\tilde{a}_{\mathrm{half}}\left( 50-a_{peak} \right)$.

^4^ In most countries we used survey-based estimates of partner age differences, but in some we calibrated partner age difference parameters to improve poor model fits to male HIV prevalence by age.

^5^ ANC calibration and variance inflation terms were fitted in every country that had corresponding ANC data (sentinel surveillance, site-level routine testing, census-level routine testing) available.

# Impact Evaluation Scenarios

For our main analyses we compared scenarios in which UNAIDS targets were reached to a counterfactual scenario in which program coverage levels remained constant from 2020 onward at 2019 levels. Programs and interventions scaled up consisted of:

- ART coverage for all PLHIV.
- Viral suppression for all PLHIV on ART.
- ART initiation before women become pregnant.
- ART initiation among pregnant women not already on ART.
- Improved retention on ART among pregnant women.
- Improved retention on ART during breastfeeding.
- Condom use in MSM, FSW, and the general population.
- Outreach programs for FSW, MSM, and PWID.
- PrEP for general and key populations.
- Voluntary medical male circumcision.
- Needle-syringe exchange programs among PWID.
- Opioid substitution therapy among PWID.
- School-based programs and sexuality education for school-aged 15-24-year-olds.

Goals ASM does not include additional inputs governing ART uptake and retention for pregnant women beyond those in AIM. Rates of mother-to-child transmission in AIM by prophylaxis status are reported elsewhere [[3](#_ENREF_3)].

Table 2 reports parameter values used in the present analysis. While Goals ASM incorporates several behavior change programs (see “Behavior change programs” above), targets were specified for only two of these: school-based prevention and sexuality education for school-aged adolescents and young adults, economic empowerment for adolescent girls and young women. The modeled effects of these programs on condom use are described below. We did not model delays in sexual debut, or reductions in numbers of sexual partners, age-disparate mixing, or experience of sexual or partner violence from these programs in the present analysis. We do not report effect size assumptions for behavior change programs that were not included in this exercise.

The UNAIDS targets directly specify condom use levels for key populations and other sexually active adults by risk strata. Condom use can be specified directly in Goals ASM, and can also be modulated by behavior change programs as described above (section “Behavior change programs”). To ensure that condom use targets were modeled precisely, we calculated the impact of key population outreach interventions required to meet condom use targets for those populations. Thus, outreach programs had country-specific impact depending on outreach targets and pre-existing outreach coverage levels in each country. To model condom use targets in the general population, we directly inputted condom use trends modified from the counterfactual scenario to account for the effects of reaching target condom use levels weighted by the proportion of people in each risk strata (see the main manuscript for descriptions of these strata). To avoid double counting the effects of condom use increases, we set the effects of general population behavior change programs on condom use to zero.

Table 2. Goals ASM parameter list and values.

| Parameter | Symbol | Value^1^ | Source |
| --- | --- | --- | --- |
| *HIV epidemiology* |  |  |  |
| Probability of HIV transmission per partnership | $\beta$ | By country | Fitted |
| HIV transmission multiplier, primary infection | $\tilde{\beta}_{P}$ | 26 | [[4](#_ENREF_4)] |
| HIV transmission multiplier, asymptomatic infection | $\tilde{\beta}_{A}$ | 1 | [[4](#_ENREF_4)] |
| HIV transmission multiplier, symptomatic infection | $\tilde{\beta}_{S}$ | 7 | [[4](#_ENREF_4)] |
| Duration of primary HIV infection | $w$ | 2.9 months | [[4](#_ENREF_4)] |
| STI prevalence | $\kappa_{s,a}^{\mathrm{STI}}(t)$ | By country | [[16](#_ENREF_16), [45](#_ENREF_45), [46](#_ENREF_46)] |
| Relative risk of HIV acquisition due to STI | $\varepsilon_{s}^{\mathrm{STI}}$ | 3 | [[17-19](#_ENREF_17)] |
|  |  |  |  |
| *Sexual behavior* |  |  |  |
| Average number of partners females have over their lifetime | $L(t)$ | By country | Fitted |
| Age when sexual partner acquisition rates peak | $a_{\mathrm{peak}}$ | By country | Fitted |
| Age by which half of lifetime sexual partners have been acquired | $a_{\mathrm{half}}$ | By country | Fitted |
| Average age difference between males and their female partners | $\mu_{m}$ | By country | [[15](#_ENREF_15)] |
| Variance in age differences between males and their female partners | $\sigma_{m}^{2}$ | By country | [[15](#_ENREF_15)] |
| Condom use at last sex | $\tilde{\kappa}_{a}\left( t \right)$ | By country | [[15](#_ENREF_15)] |
|  |  |  |  |
| *HIV programs* |  |  |  |
| Number on PMTCT through 2020 |  | By country | [[39](#_ENREF_39)] |
| Number on ART through 2020 |  | By country | [[39](#_ENREF_39)] |
| % of ART patients who are virally suppressed through 2020^2^ | $\nu_{z,b}(t)$ | By country | [[47](#_ENREF_47), [48](#_ENREF_48)] |
| Male circumcision prevalence | $\kappa_{M,a}^{\mathrm{MC}}\left( t \right)$ | By country | [[15](#_ENREF_15)] |
|  |  |  |  |
| *Biomedical intervention effects on HIV transmission* |  |  |  |
| Reduction in HIV transmission when virally suppressed on ART | $\varepsilon^{\mathrm{VS}}$ | 100% | [[6](#_ENREF_6), [12-14](#_ENREF_12)] |
| Reduction in HIV transmission from condom use | $\varepsilon^{\mathrm{Cndm}}$ | 80% | [[11](#_ENREF_11), [26](#_ENREF_26)] |
| Reduction in HIV acquisition when receiving daily oral PrEP | $\varepsilon_{s,a,1}^{\mathrm{PrEP}}$ | 70% | [[20](#_ENREF_20)] |
| - Oral PrEP efficacy | $\eta_{s,a,1}^{\mathrm{PrEP}}$ | 99% | [[20-22](#_ENREF_20)] |
| - Adherence to daily oral PrEP | $\alpha_{s,a,1}^{\mathrm{PrEP}}$ | 71% | [[20](#_ENREF_20)] |
| Reduction in HIV acquisition among circumcised males | $\varepsilon^{\mathrm{MC}}$ | 60% | [[23-25](#_ENREF_23)] |
|  |  |  |  |
| *Effects of behavior change programs in the general population*^3^ |  |  |  |
| Reduction in condom non-use from school-based prevention and sexuality education ($a=$15-24) | $\varepsilon_{s,a,j,3}^{\mathrm{BCP}}$ | See note^4^ |  |
| Reduction in condom non-use from economic empowerment ($s=$Female, $a=$ 15-24) | $\varepsilon_{s,a,j,3}^{\mathrm{BCP}}$ | See note^4^ |  |
|  |  |  |  |
| *Effects of behavior change programs in key populations*^3^ |  |  |  |
| Reduction in condom non-use from outreach ($r=\mathrm{FSW}$) | $\varepsilon_{r,a}^{\mathrm{OR}}$ | By country | Calculated |
| Reduction in condom non-use from outreach ($r=\mathrm{MSM}$) | $\varepsilon_{r,a}^{\mathrm{OR}}$ | By country | Calculated |
| Reduction in condom non-use from outreach ($r=\mathrm{FWID}$ or $\mathrm{MWID}$) | $\varepsilon_{r,a}^{\mathrm{OR}}$ | 0.0% | Assumed |
| Reduction in PWID needle sharing from outreach | $\varepsilon_{r,a,\mathrm{OR}}^{SI,1}$ | -39.5% | [[49-56](#_ENREF_49)] |
| Reduction in PWID needle sharing from needle-syringe exchange | $\varepsilon_{r,a,\mathrm{NSEP}}^{SI,1}$ | -24.0% | [[31](#_ENREF_31)] |
| Reduction in PWID needle sharing from opioid substitution therapy | $\varepsilon_{r,a,\mathrm{OST}}^{SI,1}$ | -42.4% | [[32](#_ENREF_32), [33](#_ENREF_33)] |
| Reduction in unsafe injecting behavior from outreach | $\varepsilon_{r,a,\mathrm{OR}}^{SI,2}$ | -45.3% | [[49](#_ENREF_49), [50](#_ENREF_50)] |
| Reduction in unsafe injecting behavior from needle-syringe exchange | $\varepsilon_{r,a,\mathrm{NSEP}}^{SI,2}$ | 0.0% | Assumed |
| Reduction in unsafe injecting behavior from opioid substitution therapy | $\varepsilon_{r,a,\mathrm{OST}}^{SI,2}$ | -63.0% | [[32](#_ENREF_32), [33](#_ENREF_33)] |

^1^ Country-specific values were used for parameters listed as “By country”, otherwise the same value was used in all countries where Goals ASM was used.

^2^ We used estimates of viral suppression on ART from PHIA surveys (preferred), published by UNAIDS via http://aidsinfo.unaids.org (next preferred), or a 75% default value (least preferred).

^3^ Behavior change program effects that increase condom use or reduce other behavioral risk factors are expressed as negative numbers (for example, see Equation 26).

^4^ Condom use targets for the general population were specified directly. To avoid double counting the effects of achieving condom use targets, we assumed general population behavior change programs did not further increase condom use.

REFERENCES

1. Pretorius C, Stover J, Bollinger L, Bacaër N, Williams BG. Evaluating the cost-effectiveness of pre-exposure prophylaxis (PrEP) and its impact on HIV-1 transmission in South Africa. PLoS One. 2010;5(11):e13646. doi: 10.1371/journal.pone.0013646.

2. Avenir Health. Spectrum Manual: Spectrum System of Policy Models [cited 2017 Feb. 16]. Available from: <http://avenirhealth.org/Download/Spectrum/Manuals/SpectrumManualE.pdf>.

3. Stover J, Glaubius R, Mofenson L, Dugdale CM, Davies M-A, Patten G, et al. Updates to the Spectrum/AIM model for estimating key HIV indicators at national and subnational levels. AIDS. 2019;33(Suppl 3):S227-S34. doi: 10.1097/QAD.0000000000002357.

4. Hollingsworth TD, Anderson RM, Fraser C. HIV-1 transmission, by stage of infection. J Infect Dis. 2008;198(5):687-93. doi: 10.1086/590501.

5. Quinn TC, Wawer MJ, Sewankambo N, Serwadda D, Li C, Wabwire-Mangen F, et al. Viral load and heterosexual transmission of human immunodeficiency virus type 1. N Engl J Med. 2000;342(13):921-9. doi: 10.1056/NEJM200003303421303.

6. Cohen MS, Chen YQ, McCauley M, Gamble T, Hosseinipour MC, Kumarasamy N, et al. Prevention of HIV-1 infection with early antiretroviral therapy. N Engl J Med. 2011;365(6):493-505. doi: 10.1056/NEJMoa1105243.

7. Johnson LF, Anderegg N, Zaniewski E, Eaton JW, Rebeiro PF, Carriquiry G, et al. Global variations in mortality in adults after initiating antiretroviral treatment: an updated analysis of the International epidemiology Databases to Evaluate AIDS cohort collaboration. AIDS. 2019;33(Suppl 3):S283-S94. doi: 10.1097/QAD.0000000000002358.

8. Garnett GP, Anderson RM. Balancing sexual partnerships in an age and activity stratified model of HIV transmission in heterosexual populations. IMA J Math Appl Med Biol. 1994;11(3):161-92. doi: 10.1093/imammb/11.3.161.

9. Garnett GP, Anderson RM. Factors controlling the spread of HIV in heterosexual communities in developing countries: patterns of mixing between different age and sexual activity classes. Philos Trans R Soc Lond B Biol Sci. 1993;342(1300):137-59. doi: 10.1098/rstb.1993.0143.

10. Wilson DP, Law MG, Grulich AE, Cooper DA, Kaldor JM. Relation between HIV viral load and infectiousness: a model-based analysis. Lancet. 2008;372(9635):314-20. doi: 10.1016/S0140-6736(08)61115-0.

11. Hughes JP, Baeten JM, Lingappa JR, Magaret AS, Wald A, de Bruyn G, et al. Determinants of per-coital-act HIV-1 infectivity among African HIV-1-serodiscordant couples. J Infect Dis. 2012;205(3):358-65. doi: 10.1093/infdis/jir747.

12. Bavington BR, Pinto AN, Phanuphak N, Grinsztejn B, Prestage G, Zablotska-Manos IB, et al. Viral suppression and HIV transmission in serodiscordant male couples: an international, prospective, observational, cohort study. Lancet HIV. 2018;5(8):e438-e47. doi: 10.1016/S2352-3018(18)30132-2.

13. Rodger AJ, Cambiano V, Bruun T, Vernazza P, Collins S, van Lunzen J, et al. Sexual activity without condoms and risk of HIV transmission in serodifferent couples when the HIV-positive partner is using suppressive antiretroviral therapy. JAMA. 2016;316(2):171-81. doi: 10.1001/jama.2016.5148.

14. Attia S, Egger M, Müller M, Zwahlen M, Low N. Sexual transmission of HIV according to viral load and antiretroviral therapy: systematic review and meta-analysis. AIDS. 2009;23(11):1397-404. doi: 10.1097/QAD.0b013e32832b7dca.

15. ICF. Demographic and Health Surveys (various) and AIDS Indicator Surveys (various). Funded by USAID. Rockville, Maryland: 2003-2018.

16. Looker KJ, Magaret AS, Turner KME, Vickerman P, Gottlieb SL, Newman LM. Global estimates of prevalent and incident Herpes Simplex Virus Type 2 infections in 2012. PLoS One. 2015;10(1):e114989. doi: 10.1371/journal.pone.0114989.

17. Freeman EE, Weiss HA, Glynn JR, Cross PL, Whitworth JA, Hayes RJ. Herpes simplex virus 2 infection increases HIV acquisition in men and women: systematic review and meta-analysis of longitudinal studies. AIDS. 2006;20(1):73-83. doi: 10.1097/01.aids.0000198081.09337.a7.

18. Mahiane S-G, Legeai C, Taljaard D, Latouche A, Puren A, Peillon A, et al. Transmission probabilities of HIV and herpes simplex virus type 2, effect of male circumcision and interaction: a longitudinal study in a township of South Africa. AIDS. 2009;23(3):377-83. doi: 10.1097/QAD.0b013e32831c5497.

19. Boily M-C, Baggaley RF, Wang L, Mâsse B, White RG, Hayes RJ, et al. Heterosexual risk of HIV-1 infection per sexual act: systematic review and meta-analysis of observational studies. Lancet Infect Dis. 2009;9(2):118-29. doi: 10.1016/S1473-3099(09)70021-0.

20. Fonner VA, Dalglish SL, Kennedy CE, Baggaley R, O'Reilly KR, Koechlin FM, et al. Effectiveness and safety of oral HIV pre-exposure prophylaxis (PrEP) for all populations: a systematic review and meta-analysis. AIDS. 2016;30(12):1973-83. doi: 10.1097/QAD.0000000000001145.

21. Anderson PL, Glidden DV, Liu A, Buchbinder S, Lama JR, Guanira JV, et al. Emtricitabine-tenofovir concentrations and pre-exposure prophylaxis efficacy in men who have sex with men. Sci Transl Med. 2012;4(151):151ra25. doi: 10.1126/scitranslmed.3004006.

22. Donnell D, Baeten JM, Bumpus NN, Brantley J, Bangsberg DR, Haberer JE, et al. HIV protective efficacy and correlates of tenofovir blood concentrations in a clinical trial of PrEP for HIV prevention. J Acquir Immune Defic Syndr. 2014;66(3):340-8. doi: 10.1097/QAI.0000000000000172.

23. Auvert B, Taljaard D, Lagarde E, Sobngwi-Tambekou J, Sitta R, Puren A. Randomized, controlled intervention trial of male circumcision for reduction of HIV infection risk: the ANRS 1265 Trial. PLoS Med. 2005;2(11):e298. doi: 10.1371/journal.pmed.0020298.

24. Bailey RC, Moses S, Parker CB, Agot K, Maclean I, Krieger JN, et al. Male circumcision for HIV prevention in young men in Kisumu, Kenya: a randomised controlled trial. Lancet. 2007;369(9562):643-56. doi: 10.1016/S0140-6736(07)60312-2.

25. Gray RH, Kigozi G, Serwadda D, Makumbi F, Watya S, Nalugoda F, et al. Male circumcision for HIV prevention in men in Rakai, Uganda: a randomised trial. Lancet. 2007;369(9562):657-66. doi: 10.1016/S0140-6736(07)60313-4.

26. Weller SC, Davis-Beaty K. Condom effectiveness in reducing heterosexual HIV transmission. Cochrane Database Syst Rev. 2002;(1):CD003255. doi: 10.1002/14651858.CD003255.

27. Viswasam N, Lyons CE, MacAllister J, Millett G, Sherwood J, Rao A, et al. The uptake of population size estimation studies for key populations in guiding HIV responses on the African continent. PLoS One. 2020;15(2):e0228634. doi: 10.1371/journal.pone.0228634.

28. Shannon K, Crago A-L, Baral SD, Bekker L-G, Kerrigan D, Decker MR, et al. The global response and unmet actions for HIV and sex workers. Lancet. 2018;392(10148):698-710. doi: 10.1016/S0140-6736(18)31439-9.

29. Sabin K, Zhao J, Calleja JMG, Sheng Y, Garcia SA, Reinisch A, et al. Availability and quality of size estimations of female sex workers, men who have sex with men, people who inject drugs and transgender women in low- and middle-income countries. PLoS One. 2016;11(5):e0155150. doi: 10.1371/journal.pone.0155150.

30. Bollinger LA. How can we calculate the "E" in "CEA"? AIDS. 2008;22(Suppl 1):S51-S7. doi: 10.1097/01.aids.0000327623.31856.45.

31. Aspinall EJ, Nambiar D, Goldberg DJ, Hickman M, Weir A, Van Velzen E, et al. Are needle and syringe programmes associated with a reduction in HIV transmission among people who inject drugs: a systematic review and meta-analysis. Int J Epidemiol. 2014;43(1):235-48. doi: 10.1093/ije/dyt243.

32. Nielsen S, Larance B, Degenhardt L, Gowing L, Kehler C, Lintzeris N. Opioid agonist treatment for pharmaceutical opioid dependent people. Cochrane Database Syst Rev. 2016;(5):CD011117. doi: 10.1002/14651858.CD011117.pub2.

33. MacArthur GJ, Minozzi S, Martin N, Vickerman P, Deren S, Bruneau J, et al. Opiate substitution treatment and HIV transmission in people who inject drugs: systematic review and meta-analysis. BMJ. 2012;345:e5945. doi: 10.1136/bmj.e5945.

34. Joint United Nations Programme on HIV/AIDS. Key Population Atlas: UNAIDS; 2021. Available from: <https://kpatlas.unaids.org/>.

35. Degenhardt L, Peacock A, Colledge S, Leung J, Grebely J, Vickerman P, et al. Global prevalence of injecting drug use and sociodemographic characteristics and prevalence of HIV, HBV, and HCV in people who inject drugs: a multistage systematic review. Lancet Glob Health. 2017;5(12):e1192-e207. doi: 10.1016/S2214-109X(17)30375-3.

36. Baral S, Beyrer C, Muessig K, Poteat T, Wirtz AL, Decker MR, et al. Burden of HIV among female sex workers in low-income and middle-income countries: a systematic review and meta-analysis. Lancet Infect Dis. 2012;12(7):538-49. doi: 10.1016/S1473-3099(12)70066-X.

37. Nelder JA, Mead R. A simplex method for function minimization. Compute J. 1965;7(4):308-13.

38. Eaton JW, Brown T, Puckett R, Glaubius R, Mutai KK, Bao L, et al. The Estimation and Projection Package Age-Sex Model and the R-hybrid model: new tools for estimating HIV incidence trends in sub-Saharan Africa. AIDS. 2019;33(Suppl 3):S235-S44. doi: 10.1097/QAD.0000000000002437.

39. Joint United Nations Programme on HIV/AIDS. National HIV estimates file 2020 [June 22, 2020]. Available from: <https://www.unaids.org/en/dataanalysis/datatools/spectrum-epp>.

40. UNAIDS/WHO working group on global HIV/AIDS and STI surveillance. Conducting HIV surveillance among pregnant women attending antenatal clinics based on routine programme data. Geneva: World Health Organization; 2015.

41. World Health Organization. Consolidated guidelines on HIV testing services. Geneva: World Health Organization; 2015.

42. Sheng B, Marsh K, Slavkovic AB, Gregson S, Eaton JW, Bao L. Statistical models for incorporating data from routine HIV testing of pregnant women at antenatal clinics into HIV/AIDS epidemic estimates. AIDS. 2017;31(Suppl 1):S87-S94. doi: 10.1097/QAD.0000000000001428.

43. Alkema L, Raftery AE, Clark SJ. Probabilistic projections of HIV prevalence using Bayesian melding. Ann Appl Stat. 2007;1(1):229-48. doi: 10.1214/07-aoas111.

44. Eaton JW, Bao L. Accounting for nonsampling error in estimates of HIV epidemic trends from antenatal clinic sentinel surveillance. AIDS. 2017;31(Suppl 1):S61-S8. doi: 10.1097/QAD.0000000000001419.

45. The DHS Program. STATcompiler 2021. Available from: <https://www.statcompiler.com/en/>.

46. National AIDS and STI Control Program (NASCOP). Kenya AIDS Indicator Survey 2007. Nairobi: NASCOP, 2009.

47. ICAP at Columbia University. PHIA Project: A Drop that Counts [cited 2021 March 3]. Available from: <https://phia.icap.columbia.edu/>.

48. Joint United Nations Programme on HIV/AIDS. AIDSInfo: UNAIDS; 2020 [Jul 31, 2020]. Available from: <http://aidsinfo.unaids.org/>.

49. Solomon SS, Celentano DD, Srikrishnan AK, Vasudevan CK, Murugavel KG, Iqbal SH, et al. Low incidences of human immunodeficiency virus and hepatitis C virus infection and declining risk behaviors in a cohort of injection drug users in Chennai, India. Am J Epidemiol. 2010;172(11):1259-67. doi: 10.1093/aje/kwq288.

50. Booth RE, Mikulich-Gilbertson SK, Brewster JT, Salomonsen-Sautel S, Semerik O. Predictors of self-reported HIV infection among drug injectors in Ukraine. J Acquir Immune Defic Syndr. 2004;35(1):82-8. doi: 10.1097/00126334-200401010-00012.

51. Gilbert L, El-Bassel N, Terlikbayeva A, Rozental Y, Chang M, Brisson A, et al. Couple-based HIV prevention for injecting drug users in Kazakhstan: a pilot intervention study. J Prev Interv Community. 2010;38(2):162-76. doi: 10.1080/10852351003640914.

52. Chen HT, Liang S, Liao Q, Wang S, Schumacher JE, Creger TN, et al. HIV voluntary counseling and testing among injection drug users in south China: a study of a non-government organization based program. AIDS Behav. 2007;11(5):778-88. doi: 10.1007/s10461-007-9215-x.

53. Kumar MS, Mudaliar S, Daniels D. Community-based outreach HIV intervention for street-recruited drug users in Madras, India. Public Health Reports. 1998;113(Suppl 1):58-66.

54. Chen H-T, Liao Q. A pilot study of the NGO-based relational intervention model for HIV prevention among drug users in China. AIDS Educ Prev. 2005;17(6):503-14. doi: 10.1521/aeap.2005.17.6.503.

55. Hammett TM, Kling R, Johnston P, Liu W, Ngu D, Friedmann P, et al. Patterns of HIV prevalence and HIV risk behaviors among injection drug users prior to and 24 months following implementation of cross-border HIV prevention interventions in northern Vietnam and southern China. AIDS Educ Prev. 2006;18(2):97-115. doi: 10.1521/aeap.2006.18.2.97.

56. Wei L, Chen J, Rodolph M, Beauchamp G, Mâsse B, Li R, et al. HIV incidence, retention, and changes of high-risk behaviors among rural injection drug users in Guangxi, China. Subst Abus. 2006;27(4):53-61. doi: 10.1300/j465v27n04_07.
