# Supplementary material for: Modeling the epidemiological impact of the UNAIDS 2025 targets to end AIDS as a public health threat by 2030
Source: PLoS Med. 2021 Oct 18;18(10):e1003831. doi: 10.1371/journal.pmed.1003831 (PMC8559943; doi:10.1371/journal.pmed.1003831)
Supplement: S2 Text — AEM, AIDS Epidemic Model. (DOCX) [file pmed.1003831.s003.docx]

***Supplementary Text 2. An overview of AEM and its approach to calculating program impacts***

***The AIDS Epidemic Model***

The AIDS Epidemic Model (AEM) is a process model, which means it simulates the processes of HIV transmission among the adult (age 15+) populations which are most critical in concentrated epidemics (see Figure 1):

-
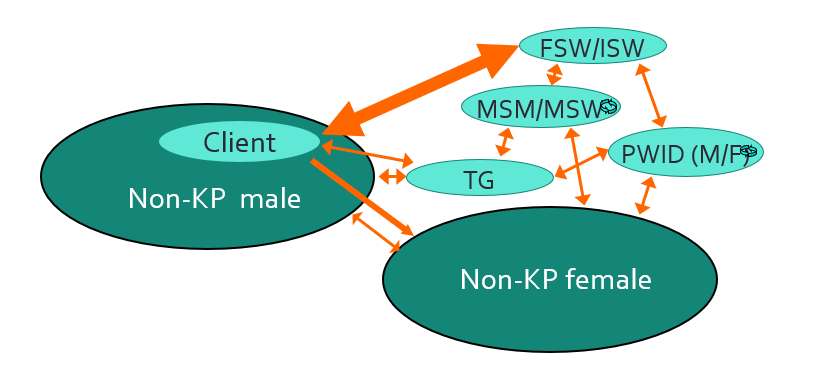
Female sex workers (FSW) and sex workers who inject (ISW)
- Clients of FSW (Clients)
- Men who have sex with men (MSM) and male sex workers (MSW)
- Transgender populations (TG)
- People who inject drugs (PWID), and
- Non-key population men and women (Non-KP men and women)

Figure 1. Groups included in AEM

The model calculates the number of new infections for each major mode of transmission in each population: vaginal sex, anal sex, and needle sharing. These calculations are done as shown conceptually in Figure 2. The needs of this calculation dictate the key inputs required, each of which must be provided as trends over time.


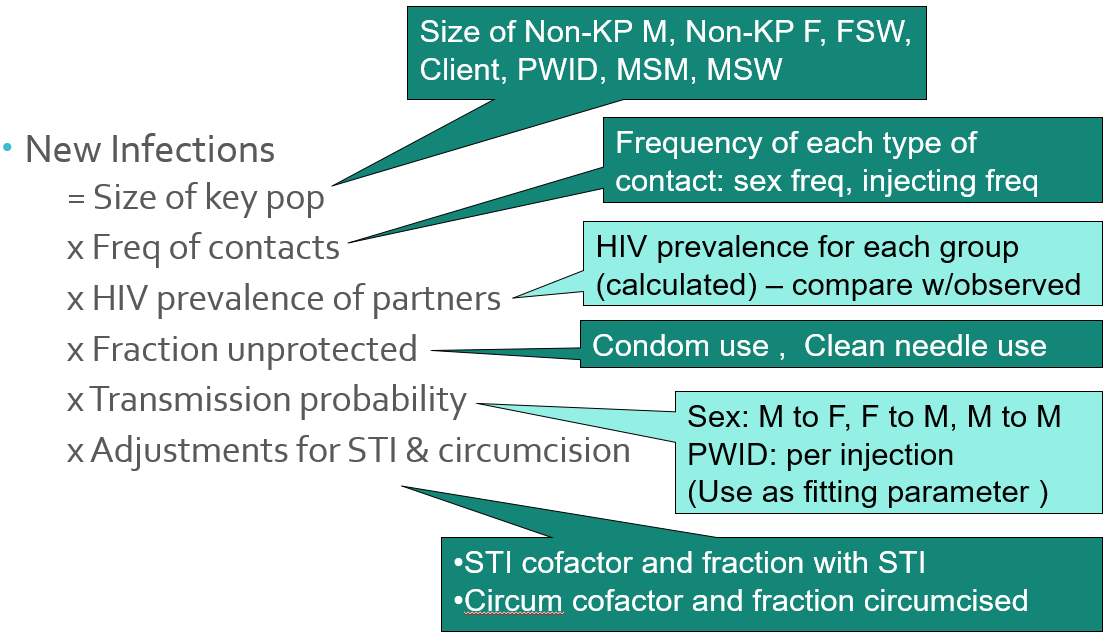


Figure 2. Conceptual model of the calculation of new infections in AEM.

These inputs, required for each population that is included in the model, are:

- Biological data trends
  - HIV prevalence, STI prevalence and male circumcision status
- Sexual behavior data trends
  - Frequency of sex, condom use with different partner types, duration of sex work/clienthood
- Injecting behavior data trends
  - Frequency of injection, level of needle sharing, duration of injecting
- Sizes of each population (entered as % of adult male or female population) and national population trends.

AEM then calculates new infections, deaths among those living with HIV and the current infections and HIV prevalence in each key population. Deaths are calculated using a built-in version of the Spectrum CD4 model, which is used by UNAIDS for calculating global estimates of deaths using mortality rates for each CD4 category, sex, and on-/off-ART status [1]. The AEM interface, shown in Figure 3 for the Thai national model in 2020 then allows the AEM team in-country to tune the model to fit the observed patterns of prevalence among the key populations. This produces a model which reflects the evolution of the epidemiological situation over time in each important population. All these inputs are stored in a single AEM Baseline workbook, an Excel file from which the model is run. The results are then read back into the same workbook, providing a complete picture of both the inputs and the outputs of the model in a single Excel workbook. Examples of these outputs are shown in Figure 4, but they include for each population in the model:

- HIV prevalence and current HIV infections over time
- Annual new HIV infections over the course of the epidemic
- HIV-related and total deaths among those living with HIV
- The number moving between key populations over time (turnover)
- Details on ART numbers, need and coverage

These are available in numerical and graphical form in the figures and tables in the Baseline workbook.


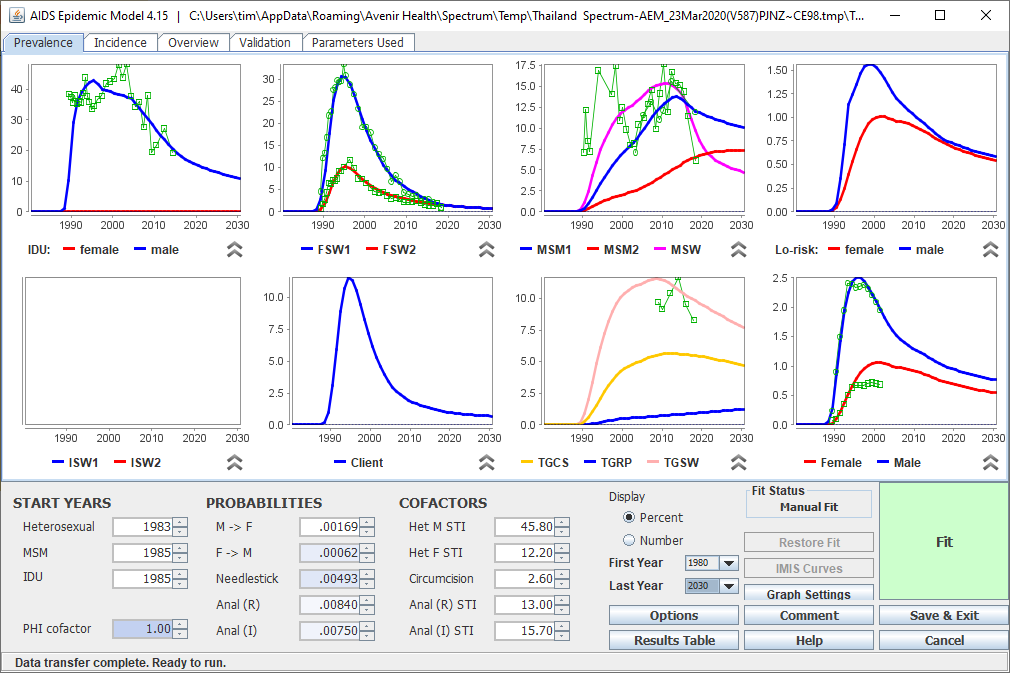


Figure 3. The AEM interface, showing the Thailand 2020 national fit.


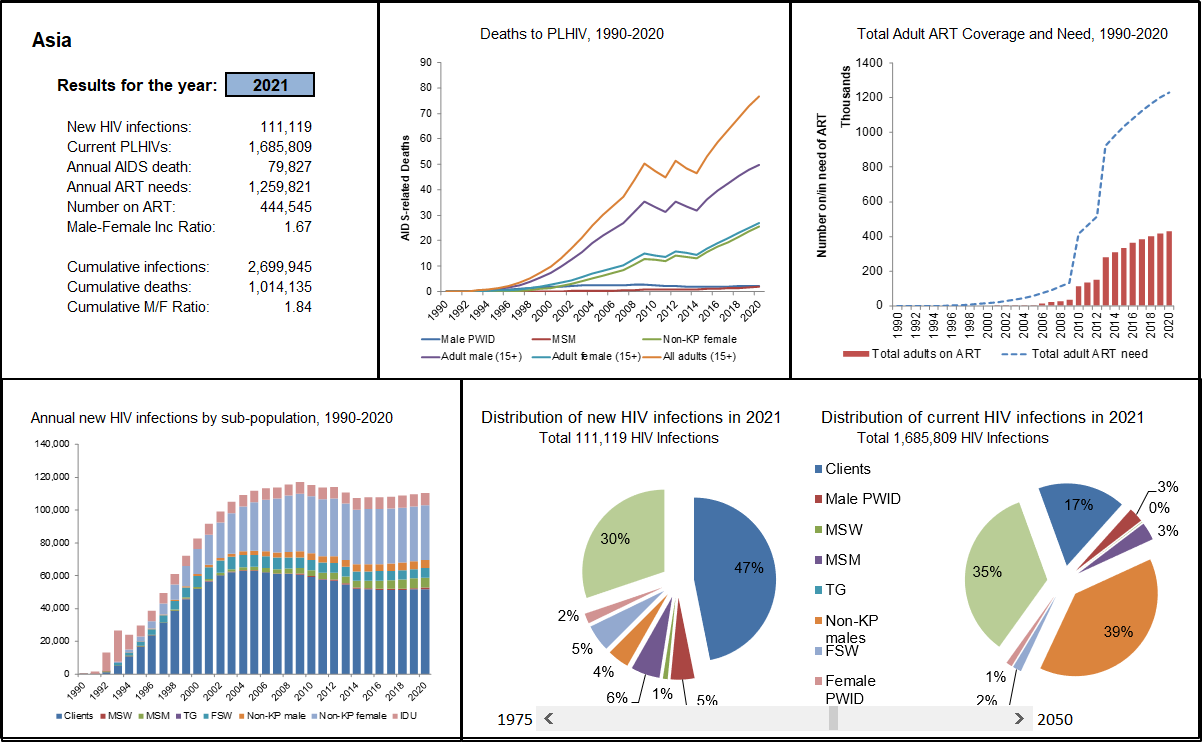


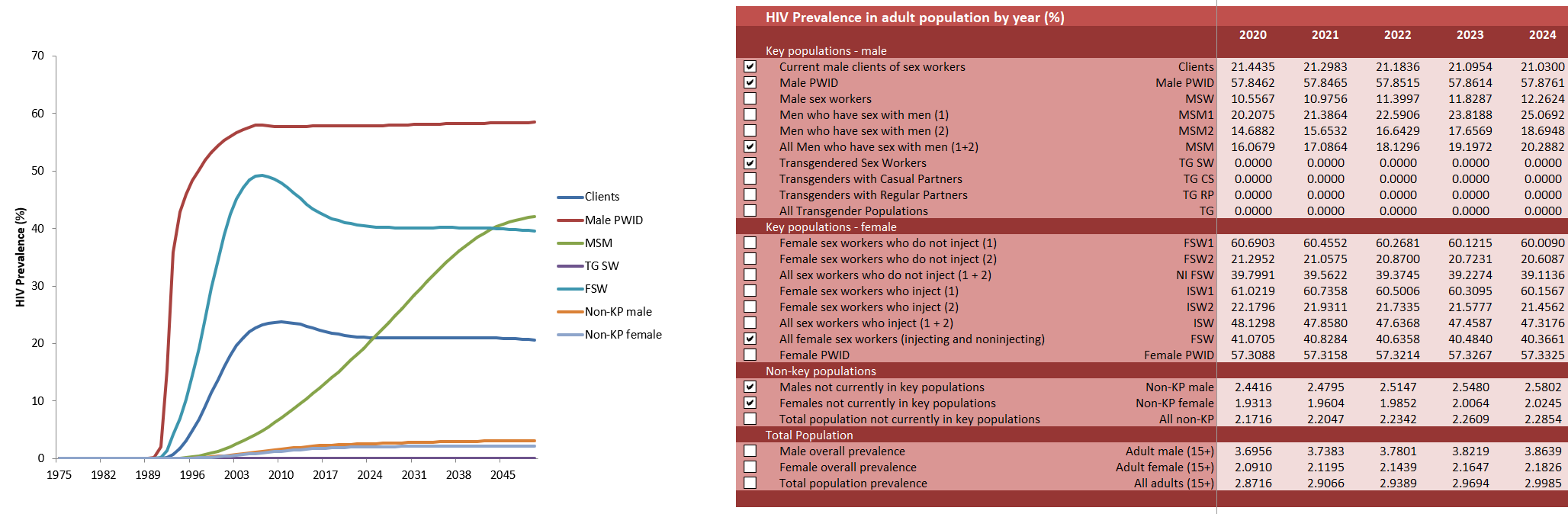


Figure 4. Examples of the outputs available in AEM

***Estimating program impacts with AEM***

The primary goal of prevention programs is to change behaviors. Because AEM is a process model, it can be used to analyze the impact of behavior changes on the course of the epidemic. In order to analyze the impact of prevention programs on behaviors, a separate AEM Intervention Workbook has been developed based on the Best Practice approach developed by UNAIDS [2]. In this approach, the user specifies the target behaviors to be achieved when coverage reaches a particular level, usually 80%, based on what programs in-country have been able to achieve in practice. A curve is then created showing the relationship between coverage and key behaviors as shown in Figure 5. The user can choose either a linear relationship or an exponential one, which yields faster initial results and diminishing returns on behavior change as coverage scales. Most countries find the latter to be more appropriate for them.


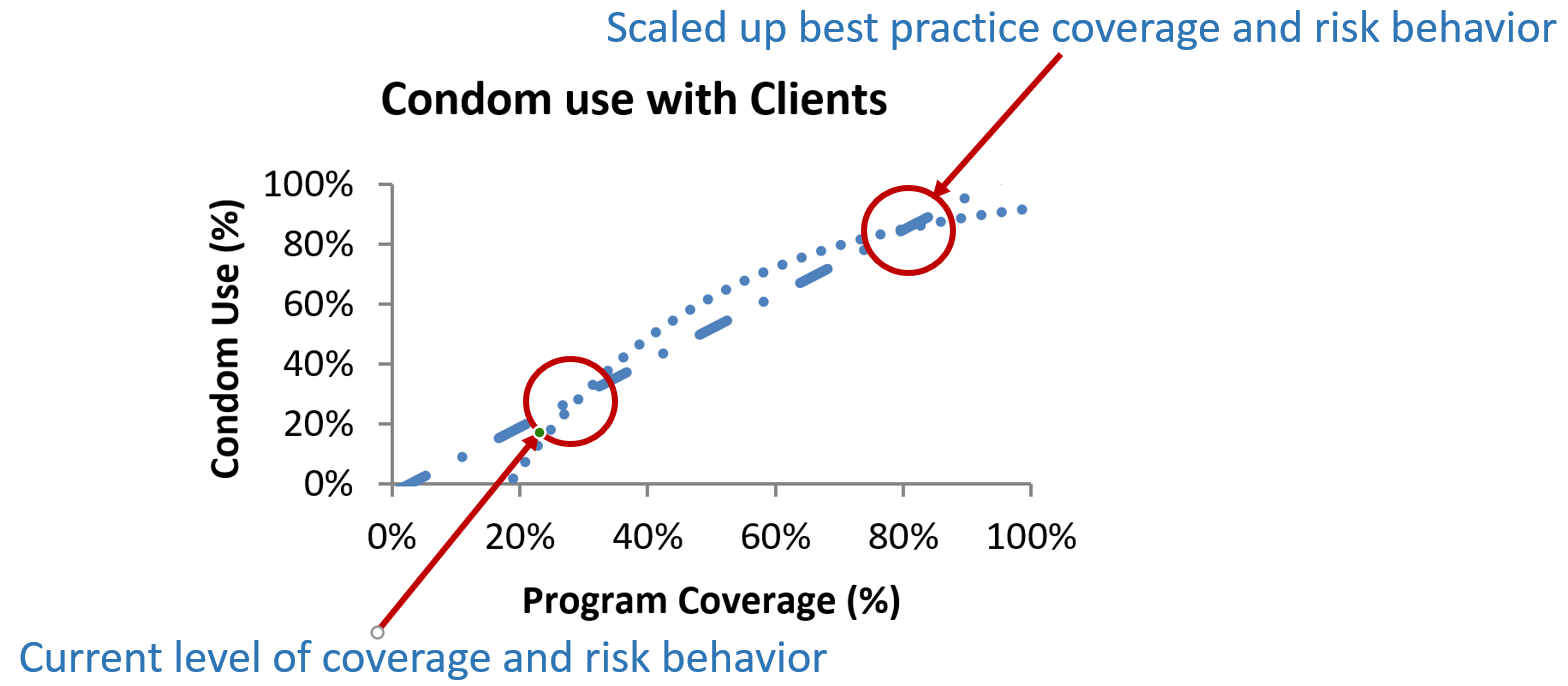


Figure 5. The behavior-coverage curve used to calculate behavior change due to program packages.

The AEM Intervention workbook is designed to allow such program coverage-behavior relationships to be defined for each key population. It also allows program unit cost data to be specified when desired to estimate overall intervention program cost. The Intervention workbook also includes ART treatment which can be specified for the overall population by sex or for each key population independently. This allows the impacts of targeted ART interventions to be explored. AEM will calculate both the prevention benefits and mortality benefits of ART. Conceptually, program effects are then calculated as shown in Figure 6.


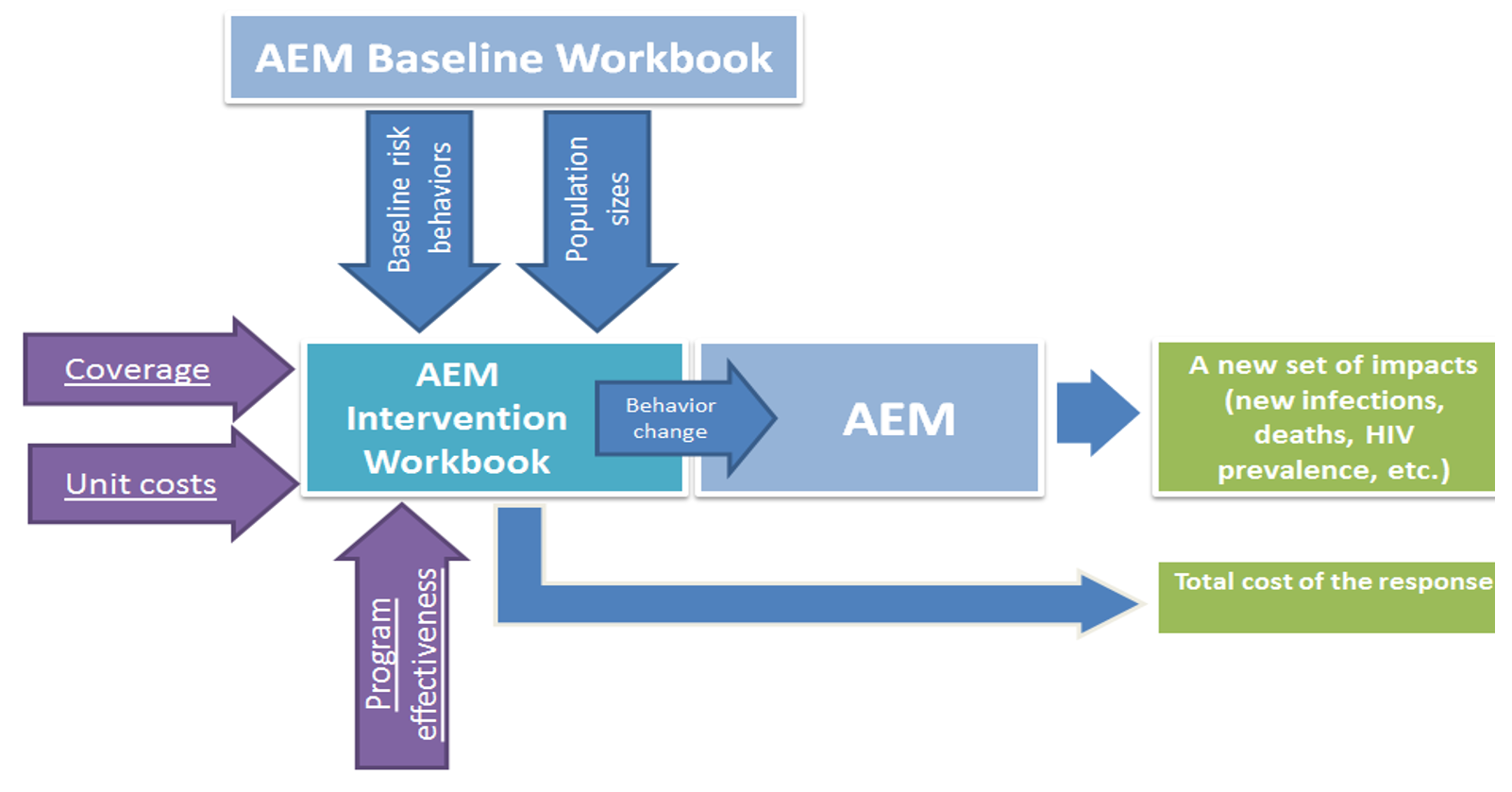


Figure 6. The AEM approach for calculating program impacts on the HIV epidemic.

The AEM Baseline and Intervention workbooks are used to create scenarios, i.e., models of the impact of alternative prevention and treatment packages on the epidemic in terms of new infections, current infections, and deaths. The final AEM workbook, the AEM Analysis workbook, then allows these scenarios to be compared in terms of their impacts. An example is shown in Figure 7. If unit costs are provided, the Analysis workbook can also estimate overall resource needs for these programs.


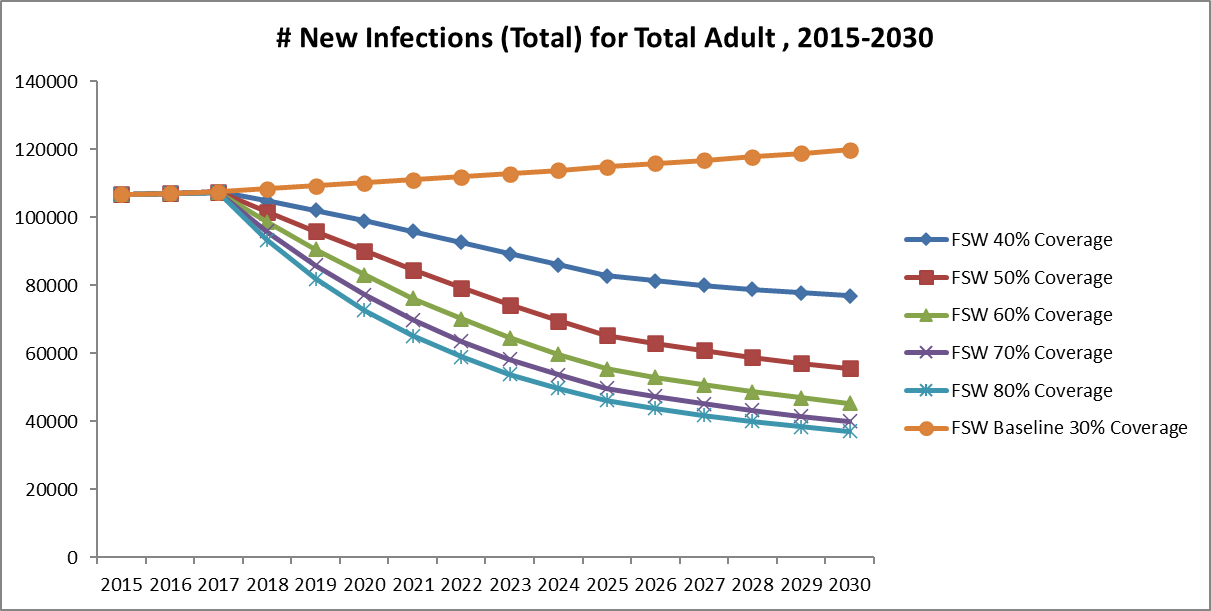


Figure 7. Example of alternative program scenarios with increasing coverage of sex worker programs.

These three tools, the Baseline, Intervention and Analysis workbooks, used together with the AEM software provide countries the ability to analyze the impacts of alternative national and sub-national responses to the HIV epidemic in concentrated epidemic settings. This provides valuable input to discussions on strategic directions and program choices for impact and cost-effectiveness.

***AEM links to Spectrum for use as a national model***

AEM can also be used as an incidence input source in Spectrum, allowing it to be used as a national model. Currently 13 countries in Asia use AEM for their national models: Bangladesh, Cambodia, Indonesia, Lao PDR, Malaysia, Mongolia, Myanmar, Nepal, Pakistan, Philippines, Sri Lanka, Thailand and Viet Nam. Of these countries 5 combine sub-national AEM projections to produce their national model: Bangladesh, Indonesia, Myanmar, Pakistan, and the Philippines. For the purposes of the work in this paper on Indonesia and Myanmar, the 2025 targets were applied to each of the sub-national projections in those countries which were then summed to give the national picture.

***References***

1. Stover J, Brown T, Marston M. Updates to the Spectrum/Estimation and Projection Package (EPP) model to estimate HIV trends for adults and children. Sex Transm Infect. 2012;88 Suppl 2:i11-6.

2. Commission on AIDS in Asia. Redefining AIDS in Asia, Crafting an Effective Response. Report of the Commission on AIDS in Asia. New Delhi: Oxford University Press; 2008. 238 p.
