# Supplementary material for: Modeling the epidemiological impact of the UNAIDS 2025 targets to end AIDS as a public health threat by 2030
Source: PLoS Med. 2021 Oct 18;18(10):e1003831. doi: 10.1371/journal.pmed.1003831 (PMC8559943; doi:10.1371/journal.pmed.1003831)
Supplement: S1 Table — (DOCX) [file pmed.1003831.s004.docx]

| Supplementary Table 1. Ranges for epidemiological parameters used for model fitting | | | |
| --- | --- | --- | --- |
|  |  |  |  |
| **Probability of HIV transmission per act** | **Median** | **Low** | **High** |
| Female to male | 0.0038 | 0.0013 | 0.011 |
| Male to female | 0.0030 | 0.0014 | 0.0063 |
|  |  |  |  |
| Anal intercourse | 0.017 | 0.003 | 0.089 |
| Multiplier | 4.47 | 2.31 | 8.09 |
| Ratio to asymptomatic stage |  |  |  |
| Primary stage | 9.2 | 4.5 | 18.1 |
| Late stage | 7.3 | 4.5 | 11.9 |
| Presence or history of genital ulcer disease | 5.3 | 1.4 | 19.5 |
|  |  |  |  |
| Source |  |  |  |
| Marie-Claude Boily, Rebecca F Baggaley, Lei Wang, Benoit Masse, Richard G White, Richard J Hayes, Michel Alary. Heterosexual risk of HIV-1 infection per sexual act: Lancet Infect Dis 2009; 9: 118–29 | | | |
|  |  |  |  |
|  | | | |
