# Supplementary material for: Modeling the epidemiological impact of the UNAIDS 2025 targets to end AIDS as a public health threat by 2030
Source: PLoS Med. 2021 Oct 18;18(10):e1003831. doi: 10.1371/journal.pmed.1003831 (PMC8559943; doi:10.1371/journal.pmed.1003831)
Supplement: S2 Table — (DOCX) [file pmed.1003831.s005.docx]

Supplementary Table 2. Fitted parameter values by country

| Country | Prob Trans | M>F multiplier | STI multiplier | MSM multiplier | Primary infection stage | Symptomatic stage |
| --- | --- | --- | --- | --- | --- | --- |
| Afghanistan | 0.00094 | 1.3 | 7.0 | 2.4 | 6.1 | 4.0 |
| Azerbaijan | 0.00074 | 1.0 | 7.0 | 2.5 | 17.5 | 3.6 |
| Bangladesh | 0.00150 | 0.8 | 2.8 | 2.0 | 14.1 | 4.0 |
| Brazil | 0.00098 | 1.6 | 5.6 | 2.0 | 8.9 | 4.0 |
| Cambodia | 0.00099 | 1.0 | 5.8 | 2.8 | 25.4 | 4.0 |
| China | 0.00105 | 1.2 | 3.5 | 2.0 | 11.0 | 4.0 |
| Colombia | 0.00106 | 1.0 | 7.0 | 1.9 | 9.2 | 4.0 |
| Cuba | 0.00092 | 1.0 | 6.0 | 2.6 | 8.1 | 4.0 |
| Djibouti | 0.00085 | 1.0 | 8.0 | 2.6 | 20.0 | 3.5 |
| France | 0.00094 | 1.4 | 5.0 | 3.1 | 7.7 | 4.0 |
| Guatemala | 0.00102 | 1.8 | 6.3 | 2.4 | 12.8 | 4.0 |
| Haiti | 0.00122 | 2.0 | 2.0 | 2.1 | 15.7 | 3.5 |
| India | 0.00131 | 0.8 | 8.0 | 2.1 | 19.0 | 4.5 |
| Indonesia | 0.00166 | 1.0 | 6.0 | 1.7 | 26.0 | 4.0 |
| Italy | 0.00071 | 1.4 | 5.0 | 4.0 | 5.2 | 4.0 |
| Jamaica | 0.00100 | 1.0 | 6.0 | 2.4 | 5.7 | 3.5 |
| Kazakhstan | 0.00101 | 1.0 | 7.0 | 1.8 | 23.3 | 4.0 |
| Kyrgyzstan | 0.00085 | 1.0 | 6.0 | 3.0 | 7.2 | 4.0 |
| Laos | 0.00115 | 1.4 | 7.0 | 1.6 | 15.0 | 4.0 |
| Lebanon | 0.00112 | 1.0 | 7.2 | 1.9 | 11.4 | 4.5 |
| Mexico | 0.00064 | 0.8 | 5.2 | 3.0 | 9.8 | 4.5 |
| Moldova | 0.00081 | 1.0 | 7.0 | 2.6 | 5.5 | 4.0 |
| Mongolia | 0.00064 | 1.0 | 5.0 | 5.5 | 4.8 | 4.0 |
| Morocco | 0.00128 | 1.0 | 7.0 | 2.0 | 6.1 | 3.6 |
| Myanmar | 0.00169 | 0.8 | 6.0 | 1.3 | 20.1 | 4.0 |
| Pakistan | 0.00068 | 1.8 | 8.4 | 2.2 | 4.3 | 3.5 |
| Paraguay | 0.00121 | 1.0 | 7.4 | 1.5 | 11.6 | 4.0 |
| Philippine | 0.00125 | 0.8 | 7.0 | 2.0 | 18.9 | 5.0 |
| PNG | 0.00070 | 1.8 | 7.0 | 2.7 | 6.4 | 5.0 |
| Russia | 0.00060 | 1.5 | 3.0 | 4.5 | 8.6 | 4.3 |
| Sudan | 0.00107 | 1.2 | 8.0 | 1.3 | 22.0 | 3.0 |
| Tajikistan | 0.00088 | 1.0 | 6.0 | 2.5 | 13.8 | 4.0 |
| Thailand | 0.00088 | 1.1 | 9.0 | 1.9 | 27.0 | 3.0 |
| Timor Leste | 0.00088 | 2.0 | 6.0 | 2.5 | 13.9 | 4.0 |
| UK | 0.00080 | 1.4 | 5.0 | 2.3 | 6.3 | 4.0 |
| Ukraine | 0.00080 | 1.4 | 7.0 | 2.0 | 8.4 | 4.0 |
| USA | 0.00065 | 1.5 | 5.0 | 2.7 | 4.2 | 4.0 |
| Viet Nam | 0.00076 | 1.0 | 7.2 | 2.3 | 20.7 | 3.2 |
