# Supplementary material for: Modeling the epidemiological impact of the UNAIDS 2025 targets to end AIDS as a public health threat by 2030
Source: PLoS Med. 2021 Oct 18;18(10):e1003831. doi: 10.1371/journal.pmed.1003831 (PMC8559943; doi:10.1371/journal.pmed.1003831)
Supplement: S3 Table — (DOCX) [file pmed.1003831.s006.docx]

# Supplemental Table 3. Effectiveness of HIV Prevention Interventions in the Goals Model

| **Intervention** | **Effectiveness** | **Indicator** | **Source** |
| --- | --- | --- | --- |
| Condom use | 78%-83% (35%-94%) | Reduction in transmission per act | [1-3] |
| Voluntary medical male circumcision | 60% (51%-64%) | Reduction in transmission per act | [4-6] |
| ART with viral suppression | 100% | Reduction in transmission per act | [7-9] |
| Oral PrEP  Overall  High adherence (>70%)  Medium adherence (40%-70%)  Low adherence (<40%)  MSM | 54% (34%-67%)  73% (61%-81%)  49% (30%-62%)  7% (0%-28%)  76% (46%-89%) | Reduction in transmission per act | [10]  [11] |
| PEP | 81% (48%-94%) | Reduction in transmission per episode | [12, 13] |
| NSEP | 42% (22%-81%) | Reduction in force of infection | [14] |
| OST | 37% (9%-57%)  60% (42%-85%) | Reduction in force of infection  Reduction in risk of HIV | [15]  [16] |
| PMTCT  On ART before pregnancy  Started ART during current pregnancy | 0.26%  0.0023%  1.40%  0.11% | Probability of peripartum transmission  Monthly probability of breastfeeding transmission  Probability of peripartum transmission  Monthly probability of breastfeeding transmission | [17] |
| Sex worker outreach | 40% (29%-50%) | OR for change in condomless sex | Author’s analysis |
| MSM outreach | 34% (10%-58%) | OR for change in condomless sex | Author’s analysis |

| Economic empowerment | 26%  0%  0% | Reduction in condomless sex  Reduction in risk behaviors (sexual debut, sexual activity, non-marital partners, alcohol use)  Reduction in condomless sex | [18]  [19]  [20] |
| --- | --- | --- | --- |
| Comprehensive sexuality education | 15%  1.34 | Reduction in condomless sex  OR for condom use | [18]  [21] |

**References**

1. Weller S, Davis K. Condom effectiveness in reducing heterosexual HIV transmission (Cochrane Review). In: The Cochrane Library, Issue 1, 2004. Chichester, UK: John Wiley & Sons, Ltd

2. Pinkerton S, Abramson PR Effectiveness of condoms in preventing HIV transmission Social Science & Medicine, May 1997, 44:9; 1303-1312, <https://doi.org/10.1016/S0277-9536(96)00258-4>

3. Hughes JP, Baeten JM, Lingappa JR, Margaret AS, Wald A, de Bruyn G et al. Determinants of Per-Coital-Act HIV-1 Infectivity Among African HIV-1 Serodiscordant Couples JID 1 February 2012; 205:358-65

4. Auvert B, Puren A, Taljaard D, Lagarde E, JoëlleTambekou-Sobngwi, RémiSitta. The impact of male circumcision on the female-to-male transmission of HIV: Results of the intervention trial: ANRS 1265.  IAS 2005: INSERM, France; 2005.

5. Bailey RC, Moses S, Parker CB, Agot K, Maclean I, Krieger JN, et al. Male circumcision for HIV prevention in young men in Kisumu, Kenya: a randomised controlled trial. Lancet. 2007; 69(9562): 643-56.

6. Gray RH, Kigozi G, Serwadda D, et al. Male circumcision for HIV prevention in men in Rakai, Uganda: a randomised trial. Lancet 2007; 369: 657–66.

7. Cohen MS, Chen YQ, McCauley M, Gamble T, Hosseinipour MC, Kumarasamy N, et al. Prevention of HIV-1 Infection with Early Antriretroviral Therapy N Engl J Med 2011; 10.1056/NEJMoa1105243

8. Attia S, Egger M, Muller M, Zwahlen M, Low N. Sexual transmission of HIV according to viral load and antiretroviral therapy:  systematic review and meta-analysis. AIDS 2009, 23:1-8.

9. Rodger, Alison J et al. Risk of HIV transmission through condomless sex in serodifferent gay couples with the HIV-positive partner taking suppressive antiretroviral therapy (PARTNER): final results of a multicentre, prospective, observational study Lancet 2019; 393: 2428-38

10. Chou R, MD; Christopher Evans, MD, MPH; Adam Hoverman, DO; Christina Sun, PhD; Tracy Dana, MLS; Christina Bougatsos, MPH; Sara Grusing, BA; P. Todd Korthuis, MD. Preexposure Prophylaxis for the Prevention of HIV Infection Evidence Report and Systematic Review for the US Preventive Services Task Force JAMA. 2019;321(22):2214-2230. doi:10.1001/jama.2019.2591

11. Huang X, Hou J, Song A, Liu X, Yang X, Xu J, Zhang J, Hu Q, Chen H, Chen Y, Meyers K and Wu H (2018) Efficacy and Safety of Oral TDF-Based Pre-exposure Prophylaxis for Men Who Have Sex With Men: A Systematic Review and Meta-Analysis. Front. Pharmacol. 9:799. doi: 10.3389/fphar.2018.00799

12. Cardo DM et al. A case-control study of HIV seroconversion in health care workers after percutaneous exposure N Engl J Med 1997 Nov 20; 337(21):1485-90

13. Irvine C. Efficacy of HIV Postexposure Prophylaxis: Systematic Review and Meta-analysis of Nonhuman Primate Studies CID 2015:60 (Suppl 3), S165

14. Aspinall E, et al. Are needle and syringe programmes associated with a reduction in HIV transmission among people who inject drugs: a systematic review and meta-analysis International Journal of Epidemiology 2014;43:235–248 doi:10.1093/ije/dyt243

15. Nielsen S, Larance B, Degenhardt L, Gowing L, Kehler C, Lintzeris N. Opioid agonist treatment for pharmaceutical opioid dependent people. Cochrane Database of Systematic Reviews 2016, Issue 5. Art. No.: CD011117. DOI: 10.1002/14651858.CD011117.pub2.

16. MacArthur GJ, Minozzi S, Martin N, Vickerman P, Deren S, Bruneau J, et al. Opiate substitution treatment and HIV transmission in people who inject drugs: systematic review and meta-analysis. BMJ 2012;345:e5945 doi:10.1136/bmj.e5945.

17. Stover J, Glaubius R, Mofenson L, Dugdale CM, Davies MA, Patten G, Yiannoutsos C. Updates to the Spectrum/AIM model for estimating key HIV indicators at national and subnational levels AIDS 2019, 33, (Suppl 3):S227-S234.

18. Mathur S, Walia M, Heck CJ, Pilgrim N, Mahapatra B. What is the effect of layered prevention interventions on HIV risk among adolescent girls in Zambia? Presented at IAS Mexico City, 23 July 2019. IAS 2019, TUAC0204

19. Nakawooya H. PEPFAR DREAMS Intervention among Adolescent Girls and Young Women in Rakai, Uganda presented at CROI 2020, Anstract OL-08, March 10, 2020. (Note that this study did find a 50% reduction in risk behaviors among AGYW 15-19 exposed to 10 Stepping Stone sessions, but no effect among 20-24 year olds.)

20. Pilgrim N, Mwapasa V, Chipeta E, Chimwaza W, Jani N, McClair T, Tenthani L, Mathur S. Evidence to Support HIV Prevention for Adolescent Girls and Young Women (AGYW) and their Male Partners, Population Council, SOAR Project, February 2020, <https://www.popcouncil.org/uploads/pdfs/2020HIV_ProjectSOAR-MalawiDREAMS.pdf>

21. Fonner VA, Armstrong KS, Kennedy CE, O’Reilly KR, Sweat MD (2014) School Based Sex Education and HIV Prevention in Low- and Middle-Income Countries: A Systematic Review and Meta-Analysis. PLoS ONE 9(3): e89692. doi:10.1371/journal.pone.0089692
