# Supplementary figures and images for: Modeling the epidemiological impact of the UNAIDS 2025 targets to end AIDS as a public health threat by 2030
Source: PLoS Med. 2021 Oct 18;18(10):e1003831. doi: 10.1371/journal.pmed.1003831 (PMC8559943; doi:10.1371/journal.pmed.1003831)

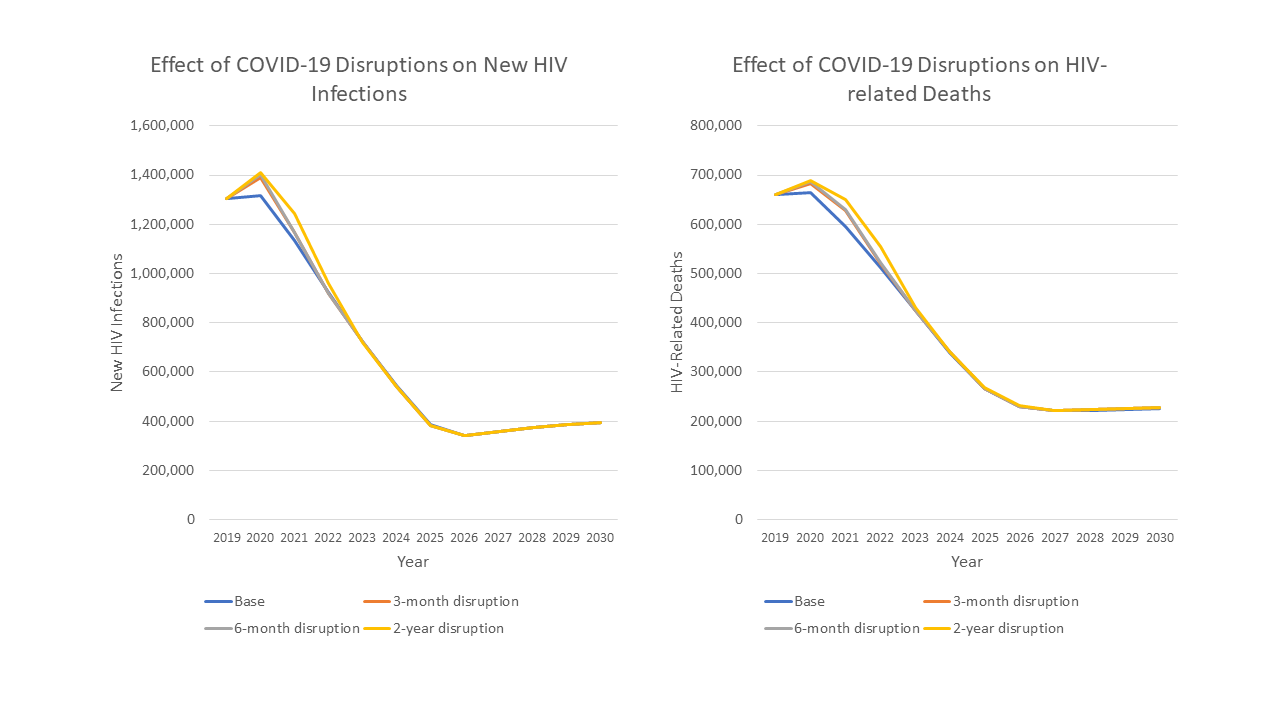

Supplement: S1 Fig — COVID-19, Coronavirus Disease 2019. (TIF) [file pmed.1003831.s008.TIF]

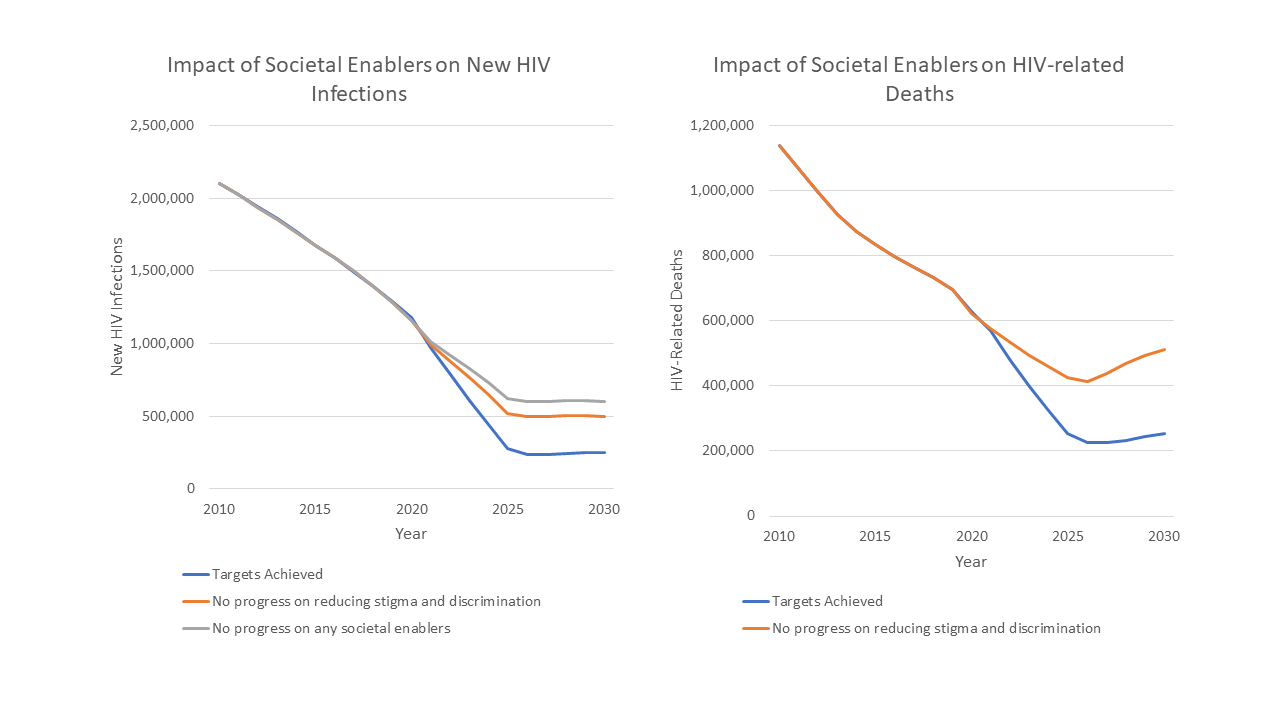

Supplement: S2 Fig — (TIF) [file pmed.1003831.s009.TIF]
