# Supplementary material for: Modeling the epidemiological impact of the UNAIDS 2025 targets to end AIDS as a public health threat by 2030
Source: PLoS Med. 2021 Oct 18;18(10):e1003831. doi: 10.1371/journal.pmed.1003831 (PMC8559943; doi:10.1371/journal.pmed.1003831)
Supplement: Attachment — Submitted filename: Response to reviewers.docx [file pmed.1003831.s010.docx]

**The Epidemiological Impact of the UNAIDS 2025 Targets for HIV" (PMEDICINE-D-21-02046R1)**

**Response to reviewers:**

Requests from the editors:

1. Please add a study descriptor to the title following a colon, e.g., "...: A modeling study".

**Response**: We have modified the title to “Modeling the Epidemiological Impact of the UNAIDS 2025 Targets To End AIDS as a Public Health Threat by 2030”.

2. You may wish to amend the title to contain the phrase "HIV control" or "AIDS elimination".

**Response**: See response to item 1.

3. Please adapt the abstract to the three-part PLOS Medicine style.

**Response**: Done

4. The final sentence of the "Methods and findings" subsection of the abstract should begin "Study limitations include ..." or similar and should quote 2-3 of the study's main limitations.

**Response**: Done

5. After the abstract, we will need to ask you to add a new and accessible "Author summary" section in non-identical prose. You may find it helpful to consult one or two recent research papers published in PLOS Medicine to get a sense of the preferred style.

**Response**: Done

6. A full point seems to be missing at line 133.

**Response**: Corrected

7. Please state, early in your Methods section, whether the study had a protocol or prespecified analysis plan, and if so attach the relevant document(s) as a supplementary file(s), referred to in the text.

**Response**: We did not have a detailed analysis plan beyond what is described in the first paragraph of the methods section. We have modified the first sentence of the Methods section to state the analysis plan.

8. Please identify a suitable checklist for your study design, which might be TRIPOD, and if available include a completed checklist as a supplementary file.

**Response**: We have added the GATHER checklist. GATHER is a WHO-recommended check list for papers publishing estimates of health indicators.

9. Please convert the "slides" into supplementary figures.

**Response**: We do not understand the reference to “slides”. Does this refer to one or more of our 6 figures?

10. To reference 1, for example, please add "UNAIDS" or another author, and specify an accessed date.

**Response**: Corrected references 1, 6 and 28.

11. Please update reference 16.

**Response**: That paper has been withdrawn. We have removed the reference.

12. It may be that reference 24 will need to be removed, unless it is available as a preprint, say.

**Response**: This has now been published. The reference has been updated.

13. Please use the journal name abbreviation "PLoS ONE".

**Response**: Corrected in reference 7, 10, 12 and 22.

Comments from the reviewers:

*** Reviewer #1:

This article was a delight to read for a number of reasons. First, it is extensive in its breadth of subject and how up to date it is. Second, it is extremely timely as many of us in the HIV community are wondering about the longer-term effects of COVID on HIV program success. Thirdly, this effort and the group of writers reflect the necessary collaboration to make meaningful estimates. The UNAIDS estimates have been a work in progress for many years and they have importantly evolved with increasingly better-quality inputs and as a result, increasingly believable.

I have several comments for the authors to consider, all of which are to increase transparency and should not be considered mandatory.

1) Many countries do not have incidence estimates either ever or that are up to date. How did you manage estimating changes in infection rates in settings with poor knowledge of incidence?

**Response**: Our analysis include estimates for 170 countries for which UNAIDS publishes estimates of incidence, PLHIV, deaths, etc. Most incidence estimates are not based on direct measurement but rather on modeling incidence by fitting the historical trends in HIV prevalence surveillance data.

2) Since condom use among clients of sex workers is typically extremely high in most settings, there is reason to believe that sex workers are less of a contributing factor to HIV among males than other risky behaviours. To what extent have you modelled sexual networks and how is defined?

**Response**: The Goals model includes eleven population groups (female sex workers, male clients of sex workers, MSM, male and female PWID, males and females with multiple partners, males and females with one partner in the last year, and males and female who are not yet sexually active. Each population group is described in terms of factors that affect transmission, including condom use, prevalence of other STIs, ART, circumcision, etc. So in settings where condom use in sex worker contacts is high, transmission between sex workers and their partners will be affected.

3) You are, no doubt, aware of the convincing evidence that men have different (generally worse) outcomes than females for access to testing, treatment, and outcomes. Morna Cornell has well summarized this. Does your model adjust for this? It historically has not.

**Response:** Yes, it does. ART use and mortality rates for those on ART are differentiated by sex. ART use by sex is based on country program data and mortality rates for those on ART are based on treatment cohort data collected and analyzed by the IeDEA Consortium.

4) Previous UNAIDS estimates have relied heavily on South Africa estimates and applied these to other countries in Africa. To what extent is borrowing occurring in the analysis (not necessarily South Africa)?

**Response**: We are no longer dependent on data from a few countries. Rates of progression in CD4 counts and HIV-related mortality of those not on ART are based on fitting models to data from national surveys, such as PHIA, and cohort studies such as the ALHPA Network and the CASCADE collaboration. For these natural history parameters, the same values are applied to all countries. HIV-related mortality for those on ART is based on analysis of cohort treatment data from a large number of countries and vary by region. Most other epi parameters (such as age and sex patterns of incidence and the effect of HIV on fertility) are derived by fitting to country-specific data.

*** Reviewer #2:

This is a well-crafted paper continuing the authors' outstanding work on informing the global HIV response by pointing out the potential impact of its ambitious, sometimes aspirational targets. The authors took great care in adding two central new aspects: the impact of (not) improving societal enablers, inasmuch as this can be estimated; and the impact of COVID-19 related interruptions. The paper fulfils its purpose fully, but this stands next to a number of shortcomings and gaps in methods and method description mentioned below.

Major comments:

1.	My main point of criticism is that it is computationally complex but conceptionally simple to tally the impact of maximizing intervention coverage towards targets that were in turn set using the same models and their estimated intervention effect sizes. That these estimates of impact have proven useful for advocacy and fundraising does not diminish the fact that, by the same token, the impact itself remains unachieved, and, one could say, unachievable. While there is a role for this paper despite this central shortcoming, one would expect the authors to spend some time describing not only the size of the underachievement under past targets, but also some of the reasons, and, more importantly, caution against the possibility of the new set of targets not being achieved (or achievable) either. This would be especially welcome given that this year marks 20 years since the first such global impact estimation- 20 years of impacts that did not come to pass, or not by the

timeline envisaged.

**Response**: These are important points. While they are outside the scope of this paper they are addressed in another paper in this collection: De Lay PR, Benzaken A, Karim QA, Aliyu S, Amole C, Ayala G, et al. (2021) Ending AIDS as a public health threat by 2030: Time to reset targets for 2025. PLoS Med 18(6): e1003649. https://doi.org/10.1371/journal.pmed.1003649. We have added this reference to the paper.

2.	One of the reasons for past underachievement is underfunding. This paper clearly has a role even without a cost envelope component, but again would be more honest if affordability could be mentioned as an issue that might well stand between the targets and the estimated impact.

***** Response**: I believe there is a separate paper in the collection on the costing and resource gap.

3.	Introduction: Summarise progress against 75% reduction targets for main parameters by 2019.

**Response**: This is done in the De Lay paper mentioned above. We have not repeated it here.

4.	Methods: The Methods section and Abstract does not specify that results were compared to Optima and AEM results for only seven countries; this is only mentioned in the Results section. Please specify this in the other sections as well. And given the size of its contribution to new infections and deaths and given that no Optima version exists for it, why were projections not also compared to a South African model, eg. Thembisa? How good is the fit of the South African GOALS model to Thembisa?

**Response**: We have modified the abstract to indicate ‘selected countries’ and the third paragraph of the Methods section to indicate that the comparison was done for seven countries for which Optima HIV and AEM have been previously used. At the time this work was done Thembisa did not have the full range of interventions needed to model the UNAIDS targets so it was not included in the validation. In other work we have compared Goals and Thembisa and found the projections and impact to be similar.

5. Given that there are explicit UNAIDS programme targets for transgender people, it would be good to have a justification for why they are not incorporated in GOALS (one would assume, just yet).

**Response**: Good point. We have added a sentence to the Goals RSM description to make it clear that we do not have a TG population group but hope to have one in the near future.

6. Line 243: "We assume that general population incidence for all other countries is below 0.3%" does not follow from there only being Naomi district-level incidence estimates for 25 countries. I would have expected how district-level differences in incidence were estimated off (available) national incidence estimates for the remaining countries, rather than a reversion to a single national-level default incidence value.

**Response**: Yes, we might have estimated proportions for other countries. But there were only five countries in the top 25 by incidence for which we did not have Naomi results (Turkmenistan, Russia, Mauritius, Belize and Jamaica). Since these are all concentrated epidemics PrEP for adolescents would not likely be cost-effective so we used these targets only for those countries for which we had Naomi results.

7.	The team is to be commended for their careful approach to the estimation of the potential impact societal enablers. However, one wonders if the literature review in reference 22 would not allow the differentiation of one of the central assumptions, the % of PLHIV with internalised stigma, by region or even country.

**Response**: We would definitely like to do this analysis with country-specific estimates of internalized stigma, because it appears to differ greatly by country. However, we could not find enough information to allow us to do that. We do hope that this may be possible as a next step in this analysis.

8.	What was the time frame (ie. which exact months and years- not just the duration) that COVID-19 related disruptions were assumed to take place?

**Response**: We assume COVID disruptions started in April 2020 and then continued for 3, 6 or 24 months. We have added this clarification to the methods section.

Minor comments:

9. Abstract: Mention full time period modelled; add the 2025 targets; mention % change for infections and deaths if societal enablers were not achieved; mention what the short-term is (for the impact of COVID-19 related disruptions).

**Response**: We have made these changes to the abstract.

10. Line 133: A full stop missing after "[10-12]".

**Response**: Added.

11. Line 197: "The program targets in the new plan" warrants a repetition of whose plan you are talking about.

**Response**: Added ‘UNAIDS’ to this sentence.

12. The two statements in line 261 onwards, "Criminalization of sex work, same sex intercourse and drug use; stigma and discrimination and gender-based violence can result in low use of prevention, testing and treatment services. Interventions to address these issues can lead to more utilization of services", need some references.

**Response**: The references are given in the following paragraphs for each enabler. We added ‘as discussed below’ to alert the reader that the references are given later.

13. Line 268: The "additional" seems misplaced here.

**Response**: There has been some progress in these areas. We wanted to make it clear that we are talking about progress beyond the current situation.

14. Line 284: Replace "might be" by "were estimated to be", and add the individual references after each estimate (even though they are also summarised in Table 5), since they seem to be directly informed by 1-2 studies each.

**Response**: Replaced ‘might be’ with ‘would be’ based on the impacts described above.

15. Line 320: "women experiencing GBV" is not a great term. Consider rephrasing.

**Response**: Replaced with ‘subject to’

16. Line 344/ 348: Please add if these total reductions would be compared to a 2010 or 2019 baseline (also in the Abstract).

**Response**: We added it in the Results. It is already in the Abstract.

17. Line 356: "because of the additional non-AIDS-related deaths to PLHIV"- perhaps rather ""because of the increase in non-AIDS-related deaths in PLHIV"? Similarly, in line 372 change "to" to "in".

**Response:** We prefer not to change this because it is not just the ‘increase’ in non-AIDS deaths but that fact that PLHIV also die from non-AIDS causes that we are trying to explain. We did change “to” to “in”.

18. Line 365: Change "continuing" to "continue".

**Response:** Thank you for spotting that. We have made the correction.

19. Page 23: You seem to not report the impact on deaths if societal enablers were not achieved, even though this is mentioned in the Abstract. Please add to the main text as well.

**Response**: Added

20. Perhaps add the finding of declining numbers of PLHIV on ART from 2023 into the Conclusion (and, if there is space, into the Abstract), as this is quite a central (and promising) finding as well.

**Response:** We have added this to the Conclusions and Abstract.

21. Line 452: Perhaps replace "or" by "unless" (and adjust the rest of the sentence).

**Response**: Done

*** Reviewer #3:

This study reports on efforts to use mathematical models to estimate the impact of achieving UNAIDS program targets for 2025, with the goal to eliminate AIDS as a public health threat by 2030.

Comments:

1. "The main results presented here were produced using one or other of two different versions of the Goals model: the age-structured version of the model, Goals ASM, was applied to 39 countries from sub-Saharan Africa while the risk-structured version, Goals RSM, was used for 38 countries from other regions." Can the authors please clarify why different approaches were applied here?

**Response:** We expanded this to read: “The main results presented here were produced using one of two different versions of the Goals model: for 39 countries in sub-Saharan Africa with generalized epidemics, where the age-pattern of sexual contact drives the epidemic, the age-structured version of the model, Goals ASM, was used while for 38 countries from other regions with concentrated epidemics, characterized by HIV transmission among key populations and their partners, the risk-structured version of the model, Goals RSM, was used.”

2. "Results from the Goals models were validated by comparing with outputs for select countries from two other models, Optima HIV, and the AIDS Epidemic Model (AEM)." Can the authors also please expand on this text, clarifying why only select countries were compared?

**Response**: These were countries where Optima and AEM had already been used. We expanded the sentence to read: “Results from the Goals models were validated by comparing with outputs from two other models, Optima HIV, and the AIDS Epidemic Model (AEM), in seven countries for which those models have been used for strategic planning.”

3. "The ranges used for the epidemiological parameters and the fitted values by country are provided in Supplementary Tables 1 and 2." The model parameters listed in Supplementary Table 2 (Fitted parameter values by country) appear to be fixed constants. Did the authors consider accounting for variability and uncertainty in there parameter estimates within the models?

**Response**: The parameter values shown in Supplementary Table 2 were derived by fitting to the epidemic in each country. There are uncertainty ranges associated with each fitted value which are used to derive uncertainty ranges for the output. We have modified the text to make this clear.

4. "The model is available for download free of charge from the Avenir Health web site as a module in the Spectrum software at <https://avenirhealth.org/software-spectrum.php>." The authors have provided a useful and impactful resource, demonstrating good practice.

**Response**: Thank you

5. "This section describes how we estimated the impact of these three societal enablers. This approach was informed by a literature review that identified interventions with evidence of impact on each of the societal enablers and/or impact on HIV behaviors or outcomes [19]. " Did the authors consider running any sensitivity analyses on the estimated value of these societal enablers?

**Response**: We did not conduct sensitivity analysis on these calculations since the number of studies available was so small we felt that the largest uncertainty was whether these studies could be applied to all countries rather than the uncertainty intervals of the effect sizes derived from the studies. However, we did also analyze the effects of societal enablers with an entirely different approach, structural equation modeling, which is described in another paper in this collection.

6. Of note, in Slide 13 there is no data plotted for the scenario of 'no progress' within the AIDS related deaths?

**Response:** For the impact on new infections we had studies that allowed us to look at the impact of reducing stigma and discrimination as well as access to justice and gender-based violence so we have three lines for new infections (targets achieved, no progress on stigma, no progress on any social enabler), but for AIDS-related deaths we only had studies of the effects of stigma on ART uptake a continuation so only show two lines (targets achieved and no progress on stigma).

7. In general, can the authors please quantify and communicate the uncertainty surrounding point estimates attained from each model throughout the results section, as well as in all figures?

***** Response:**

8. Please can the authors label the y axis in all figures?

**Response:** In each figure the title indicates the values plotted on the y axis.

9. Overall, this is a well written manuscript with a clear and concise description of each type of model.

**Response: Thank you**

10. The mathematical formulation would be welcome as supplementary material.

**Response**: The descriptions of the Goals RSM, Optima and AEM models include references to papers that provide the mathematical formulas. For the Goals ASM model, the formulas are presented in the Supplementary Text.

*** Reviewer #4:

This is a useful high-level modeling analysis that led to target setting to achieve the updated UNAIDS 95/95/95 goals.

I still have several concerns. Specifically, I am missing the following:

1. Commentary on the feasibility of each of the different goals/targets (aside from the 'softer' targets, which were discussed to some degree)

**Response:** That commentary is provided in another paper in this collection (5.	De Lay PR, Benzaken A, Karim QA, Aliyu S, Amole C, Ayala G, st al. (2021) Ending AIDS as a public health threat by 2030: Time to reset targets for 2025. PLoS Med 18(6): e1003649. <https://doi.org/10.1371/journal.pmed.1003649>). We have added a reference to it.

2. Given lines 51-54: A critical self-assessment of the value in modeling in goal setting: how did modeling fit into the process of target setting? What are the benefits and drawbacks of this approach?

**Response:** We have added a description at the end of the Introduction of the role of modeling in the overall process to develop the plan.

3. Modeling has been used in the past in UNAIDS target settings: what was learned from previous processes and updated consequently? What has modeling gotten wrong previously? How has modeling to improve guidelines improved?

**Response:** Much of this is discussed in the De Lay paper in the same collection (cited above). We have added a new second paragraph to the Conclusions section to describe some of the improvements in this round of modeling.

4. A critical assessment and discussion of the drawbacks of the Goals model - just because the model is in line with results from Optima and AEM doesn't make the results inherently correct.

**Response**: We added the most important additional limitations to the third paragraph of the Conclusions section.

-Presumably, the people remaining unreached within each of the targets/goals may differ from those who have already been reached. Are those reached with an intervention assumed to be the same (in terms of risk of onward infection) to those who are not reached within each of the targets? The remaining populations not targeted by different interventions may possibly disproportionately contribute to onward infections. If this is the case, how are the results affected?

**Response**: This is a good point. It could affect the results either way. To some extent this dynamic is captured in the studies that report effect sizes since they do measure differences in incidence or behaviors in the intervention and control groups. So by using those effect sizes we are compensating for this dynamic.

-Sensitivity analyses: based on the uncertainties surrounding the inputs and expected impact of various interventions, I would expect a robust sensitivity analysis

**Response**: The purpose of this analysis was not to estimate the impact of a specific set of coverage targets but to estimate what would be required to achieve the overall goals of 90% reduction in new infections and AIDS deaths. Theoretically there could be multiple ways to achieve those goals. But since most of the coverage targets are at maximum feasible levels, there are few practical alternative scenarios. We have added uncertainty analysis to the Results as described above and added a sub-section on ‘Uncertainty’ to the ‘Methods’ section.
