# Supplementary material for: Modeling the epidemiological impact of the UNAIDS 2025 targets to end AIDS as a public health threat by 2030
Source: PLoS Med. 2021 Oct 18;18(10):e1003831. doi: 10.1371/journal.pmed.1003831 (PMC8559943; doi:10.1371/journal.pmed.1003831)
Supplement: Attachment — Submitted filename: Response to editors and reviewers 24sep21.docx [file pmed.1003831.s011.docx]

**Modeling the Epidemiological Impact of the UNAIDS 2025 Targets to End AIDS as a Public Health Threat by 2030**

Response to Editors and reviewers: September 24, 2021

1. The title needs a study descriptor, and we suggest modifying the title to: "Estimating the Epidemiological Impact of the UNAIDS 2025 Targets to End AIDS as a Public Health Threat by 2030: A Modeling Study".

We do not want to add ‘A Modeling Study’ to the end of the title since that would imply that this work was undertaken as a separate study after the targets were developed. In fact, this work was part of process of developing the targets and strategic plan. We have changed the title the start ‘Modeling the Epidemiological …’ rather than ‘Estimating the Epidemiological …’. We hope that is acceptable.

2. In the abstract, an additional sentence or two are needed early in the "Methods and Findings" subsection to explain the methodology, including what the "Goals model" is.

OK. We have added a couple of sentences to provide more information on the Goals model and the process.

3. At line 91, please adapt the text to note that the present paper is part of a PLOS Medicine Collection around the UNAIDS targets.

We have added the following sentence “Papers describing the process and background research that contributed to the strategy development are available in the PLoS Medicine special collection ‘UNAIDS HIV Targets’ (<https://collections.plos.org/collection/unaids-hiv/>).”

4. At line 499, please make the section heading "Discussion".

Done

5. Noting references 14 & 24, please list 6 author names in reference citations, followed by "et al.".

Done

6. Noting reference 29, please ensure that all citations have full access details.

Done

7. Thank you for including the GATHER checklist: please rename this "S1_GATHER_Checklist" or similar and refer to it as such early in the Methods section (main text).

Done

8. We suggest providing a list of supplementary files at the end of the paper.

Done

9. Are we right in thinking that the files labeled "slide 12" and "slide 13" correspond to the supplementary figures? Please rename the files as appropriate if so.

Done

10. Please ensure that all figures have labeled axes.

In all figures the title describes that vertical axis and the horizontal axis is years.

Comments from academic editor:

The authors should add a response to reviewer 2's comment 3.

Comments from Reviewers:

*** Reviewer #1:

11. The authors have adequately addressed all points I consider to be important.

Thanks

*** Reviewer #2:

Thanks for the careful revisions to your paper. Almost all my comments have been sufficiently taken into account, with the exception of the following:

12. - Comments 1, 2 and 3: It is good to know that other papers cover the aspects mentioned, but very few people will read the entire special issue. Please add a short summary of each of these aspects with a reference to the other papers where needed.

Added information on the actual achievements at the end of the first paragraph of the introduction.

13. - Comment 6: Please add the details on how many countries you had Naomi estimates for into the paper.

We do mention that these estimates were available for 25 countries.

14. - Comment 14: Please still add the individual references after each estimate in Line 284 and following.

These are shown in Table 5.

15. - Comment 15: I did not want to be prescriptive, but the point here was to move away from an active term describing women subjected to GBV, given that they most often don't have much choice in the matter. If I may make a suggestion, however, perhaps replace the term in Line 320 by exactly that, "women subjected to GBV".

Yes, that change has already been made.

*** Reviewer #3:

16. The authors have satisfactorily responded to each comment in turn, and have strengthened the reporting of the study by more clearly presenting and discussing uncertainty.

Thank you

*** Reviewer #4:

17. The authors have addressed all of my concerns.

Thank you
